# Admission TRIAGES score for unfavorable functional outcome after traumatic brain injury: association, prognostic performance, and comparison with the Glasgow coma scale

**DOI:** 10.3389/fneur.2026.1963243

**Published:** 2026-10-02

**Authors:** Weicheng Shi, Liang Xing, Jianyang Wu, Zhiwei Wang, Zhengrong Zheng, Rongsi Yang

**Affiliations:** 1 Department of Neurosurgery, ShiShi Municipal Hospital, Quanzhou, China; 2 Department of Neurosurgery, Hospital of Traditional Chinese Medicine, Chongqing, China; 3 Department of Thyroid Surgery, Second Affiliated Hospital of Fujian Medical University, Quanzhou, China

**Keywords:** functional outcome, Glasgow outcome scale, prognosis, risk stratification, traumatic brain injury, TRIAGES

## Abstract

**Background:**

The Trauma Rating Index in Age, Glasgow Coma Scale, Respiratory rate, and Systolic blood pressure (TRIAGES) is a simple trauma score that has shown prognostic value for short-term mortality in traumatic brain injury (TBI), but its relationship with functional outcome remains insufficiently characterized.

**Methods:**

We conducted a retrospective single-center cohort study of adults with TBI admitted between June 2017 and May 2026. The primary exposure was the TRIAGES score calculated using age and the first GCS, respiratory rate, and systolic blood pressure values recorded at initial emergency department presentation before any in-hospital therapeutic intervention. Admission GCS was defined as the GCS value obtained at this same initial assessment. The primary outcome was unfavorable functional status at hospital discharge, defined as a discharge Glasgow Outcome Scale (GOS) score of 1–3. Multivariable logistic regression, restricted cubic spline analysis, receiver operating characteristic (ROC) analysis, calibration, Brier score, decision curve analysis, bootstrap internal validation, sensitivity analyses, and subgroup analyses were performed.

**Results:**

Among 753 patients, 181 (24.0%) had an unfavorable functional outcome. Each 1-point increase in TRIAGES was independently associated with higher odds of unfavorable functional status after adjustment for sex, injury mechanism, number of non-reactive pupils, and Rotterdam CT score (adjusted odds ratio 2.81, 95% confidence interval 2.29–3.45; *p* < 0.001). The association was approximately linear (P for non-linearity = 0.366). TRIAGES alone achieved an area under the ROC curve (AUC) of 0.905 compared with 0.890 for admission GCS alone (ΔAUC = 0.015, 95% CI 0.001–0.029; paired DeLong *p* = 0.034). Within this cohort, a Youden index-derived threshold of TRIAGES ≥3 yielded a sensitivity of 0.956 and specificity of 0.701. Bootstrap internal validation showed an optimism-corrected AUC of 0.905, and the findings remained consistent across multiple sensitivity analyses.

**Conclusion:**

Higher TRIAGES scores at initial emergency presentation were independently associated with unfavorable functional status at hospital discharge in adults with TBI. TRIAGES showed good prognostic performance and a small within-cohort increase in discrimination relative to admission GCS alone while retaining the practical advantage of using only four routinely available variables. Prospective multicenter studies with external validation and standardized long-term functional outcomes are warranted.

## 1 Introduction

TBI remains a major global health problem and is an important cause of mortality and long-term disability across all age groups (1, 2). Despite advances in trauma systems and neurocritical care, clinical outcomes after TBI remain highly heterogeneous, ranging from complete functional recovery to severe disability or death (2). Because a substantial proportion of survivors experience persistent neurological and functional impairment, mortality alone does not fully capture the clinical burden of TBI. Early prognostic assessment is therefore clinically relevant, particularly at the time of hospital admission, when readily available patient characteristics can provide important information on subsequent functional outcome and support early risk stratification and clinical decision-making (2, 3). Accordingly, simple and reliable tools that can identify patients at increased risk of unfavorable functional outcomes soon after presentation remain clinically valuable.

The Glasgow Coma Scale (GCS) is the most commonly used bedside instrument to determine the severity of acute brain injury due to its simplicity, speed, and widespread familiarity (4). Alone, GCS assesses only neurological responsiveness and prognosis is more accurately determined when GCS is considered with other baseline variables such as age, pupillary reactivity, physiological disturbances, and neuroimaging abnormalities (3, 4). While prognostic models such as IMPACT and CRASH use a number of these factors and provide estimates for mortality and unfavorable functional outcome, their utility is limited during the initial phase of emergency evaluation due to greater data requirements (3, 5). The TRIAGES score combines age, GCS, respiratory rate, and systolic blood pressure (SBP) into an easily calculated 0–14 score obtained during initial emergency department evaluation without the need for laboratory or neuroimaging data (5).

Evidence regarding the application of TRIAGES in TBI has begun to emerge. Jiang et al. evaluated 1,156 patients with isolated acute TBI and found that TRIAGES showed good discrimination for 24-h in-hospital mortality, with prognostic performance comparable to that of the GCS and Revised Trauma Score (6). A subsequent study from the same group focused on 982 patients aged ≥65 years with isolated TBI and similarly demonstrated that TRIAGES was useful for predicting 24-h mortality, with discrimination comparable to or slightly better than that of the GCS (7). However, both studies focused on very early mortality in isolated TBI, and the latter was restricted to older adults. Consequently, evidence regarding the association between TRIAGES and functional outcomes across a broader adult TBI population remains limited, particularly for functional status assessed at hospital discharge.

Because GCS is itself a component of TRIAGES, comparison with admission GCS should be interpreted as an assessment of incremental prognostic information rather than as a head-to-head comparison between two independent competing tools. Accordingly, whether incorporating age, respiratory rate, and SBP within the TRIAGES framework provides additional prognostic information beyond admission GCS remains incompletely characterized. This study therefore investigated the association between the TRIAGES score assessed at initial emergency department presentation and unfavorable functional status at hospital discharge in adults with TBI. The present research further examined the dose–response relationship between TRIAGES and outcome, evaluated its discrimination, calibration, and clinical utility, and quantified its incremental prognostic performance relative to admission GCS and models based on the four original TRIAGES variables. Finally, internal validation, sensitivity analyses, and prespecified subgroup analyses were performed to assess the robustness of the findings.

## 2 Materials and methods

### 2.1 Study design and population

This retrospective, single-center observational cohort study was conducted at Shishi Municipal Hospital. Consecutive patients recorded in the institutional TBI database between 1 June 2017 and 31 May 2026 were screened for eligibility. The inclusion criteria were as follows: (1) a clinical diagnosis of acute TBI documented during the index hospitalization and registration in the institutional TBI database within the study period; (2) age >18 years at the index admission; (3) an initial emergency-department assessment with baseline information permitting derivation of the TRIAGES score according to the prespecified data-processing and missing-data procedures; (4) a valid GOS score of 1–5 assessed at hospital discharge from the index hospitalization. The exclusion criteria were as follows: (1) age ≤18 years or missing age; (2) missing or invalid discharge GOS. The age threshold of >18 years was a study-specific eligibility criterion and should not be interpreted as the lower boundary of the original TRIAGES age-scoring algorithm. Of the 810 patients remaining after application of age criterion, 57 were excluded because discharge GOS was unavailable or invalid. Charts were reviewed to confirm that these patients had either been transferred to another hospital or discharged against medical advice by patient/family upon refusal of continued treatment at our institution. Functional outcomes after this point were unknown. No additional restrictions were imposed according to admission GCS severity or the presence of associated extracranial injury, allowing the primary cohort to reflect the broad clinical spectrum of adult TBI encountered in routine practice. Duplicate hospitalization identifiers were reconciled before eligibility assessment, with one record retained per hospitalization identifier; duplicate reconciliation was considered a data-quality procedure rather than an eligibility criterion.

### 2.2 Data collection

Demographic, physiologic, neurologic, imaging, injury-related, and laboratory variables were obtained from the institutional TBI database and corresponding hospitalization records. Baseline variables included age, sex, body temperature, respiratory rate, heart rate, systolic and diastolic blood pressure, admission GCS score, bilateral pupillary reactivity, number of non-reactive pupils, Rotterdam CT score (8), injury mechanism, white blood cell count, absolute neutrophil count, absolute lymphocyte count, absolute monocyte count, red blood cell distribution width-coefficient of variation, platelet count, blood glucose, serum potassium, and fibrinogen. Injury mechanism was categorized as traffic accident, fall from height, ground-level fall, or other. Prespecified data-quality checks were performed before statistical modeling; structurally invalid or clearly implausible values were treated as missing rather than automatically winsorized.

### 2.3 TRIAGES score and outcome definition

The TRIAGES score was the prespecified index exposure. TRIAGES scores were computed using the originally proposed scoring algorithm published by Shiraishi et al., without adjustment of the component weights or score thresholds. Age, GCS, respiratory rate, and SBP were allotted 0–2, 0–6, 0–2, and 0–4 points, respectively, for a maximum possible score of 0–14, with higher scores reflecting more physiologic and neurologic compromise. Consistent with the original TRIAGES algorithm, age was scored as 16–54 years (0 points), 55–74 years (1 point), and ≥75 years (2 points). The lower boundary of 16 years is part of the published scoring algorithm. Because eligibility for the present study was restricted to patients aged >18 years, patients aged 16–18 years were not included; eligible participants aged >18 to 54 years were assigned 0 age points. No TRIAGES component weights or category thresholds were modified. Detailed point assignments, boundaries, and implementation specifications are included in Supplementary Material S2 (5). In the present study, TRIAGES was calculated using age and the earliest GCS, respiratory rate, and SBP values documented immediately after arrival at the emergency department and before any therapeutic intervention initiated at the hospital. Admission GCS was defined as the first neurological assessment documented after arrival, whereas respiratory rate and SBP were the first recorded vital signs during the same initial emergency assessment. Thus, the TRIAGES score and admission GCS comparator were anchored to the same early in-hospital assessment. These measurements represent the earliest available in-hospital values; prehospital interventions or exposures that may have affected these measurements could not be reliably ascertained. Component values were assigned to the prespecified TRIAGES categories using the original boundary definitions. Missing or structurally invalid component values were not partially scored; they were handled according to the study missing-data procedure, and TRIAGES was subsequently re-derived. A single recorded respiratory-rate value of 0 was treated as missing in the primary analysis and was retained and scored according to the original algorithm in a sensitivity analysis. The primary outcome was unfavorable functional status at hospital discharge, assessed using the five-category GOS: 1, death; 2, persistent vegetative state; 3, severe disability; 4, moderate disability; and 5, good recovery. The association between TRIAGES and unfavorable discharge GOS was assessed using logistic regression (9). Consistent with established TBI studies, GOS 1–3 was classified as an unfavorable functional outcome and GOS 4–5 as a favorable outcome. The discharge GOS from the index hospitalization was used and was not imputed. Sensitivity analyses additionally considered mRS 3–6, ordinal GOS 1–5, and GOS 2–3 versus (vs.) 4–5 after excluding deaths (10).

### 2.4 Missing data

Missing covariate data were handled using multiple imputation by chained equations under a missing-at-random assumption conditional on the variables included in the imputation model. Continuous variables were imputed by predictive mean matching with five nearest donors, whereas categorical variables with missing values were imputed using multinomial logistic regression. After 10 burn-in cycles, 20 completed datasets were retained at two-cycle intervals from 50 chained-equation cycles (random seed 20,260,806). All non-derived study variables, including the outcome, were available as predictors in the conditional imputation models, whereas patient and source-row identifiers were excluded. TRIAGES and the number of non-reactive pupils were re-derived in each completed dataset. Regression coefficients and covariance matrices were combined using Rubin’s rules. Complete-case and single-imputation analyses were performed as sensitivity analyses.

### 2.5 Statistical analysis

Continuous variables are presented as mean ± standard deviation (SD) or median [interquartile range], as appropriate, and categorical variables as counts and percentages. Baseline characteristics were compared between favorable and unfavorable discharge GOS groups using Welch’s *t*-test, the Mann–Whitney U test, or Pearson’s chi-squared test, as appropriate. Standardized mean differences were additionally reported.

The association between the TRIAGES score at initial emergency department presentation and unfavorable functional status at hospital discharge was assessed using logistic regression. TRIAGES was prespecified as the primary predictor and modeled continuously, with odds ratios (ORs) and 95% confidence intervals (CIs) reported per 1-point and per 1-SD increase. Model 1 was unadjusted. Model 2, the prespecified primary adjusted model, included sex, injury mechanism, number of non-reactive pupils, and Rotterdam CT score. Model 3 additionally included blood glucose, white blood cell count, platelet count, and fibrinogen. Because age, admission GCS, respiratory rate, and SBP are components of TRIAGES, they were not simultaneously entered as separate covariates in the primary TRIAGES association models.

Potential non-linearity was assessed using restricted cubic splines (RCS). For TRIAGES, three knots were placed at the 10th, 50th, and 90th percentiles of its distribution (scores 0, 2, and 5), with TRIAGES = 2 as the reference. Overall association and non-linearity were assessed using pooled Wald tests. Rotterdam CT score showed evidence of a non-linear relationship with outcome and was therefore modeled using a three-knot RCS with knots at scores 1, 2, and 5 in Models 2 and 3. Prognostic performance was evaluated using ROC curves, AUC, calibration, Brier score, and decision curve analysis (DCA). For the TRIAGES-alone model, an exploratory operating threshold was identified within the study cohort using the Youden index, with the corresponding sensitivity and specificity reported. This data-derived threshold was evaluated in the same cohort in which it was selected and was not considered a clinically validated cutoff. Five prespecified prognostic specifications were compared: admission GCS alone, TRIAGES alone, the four original TRIAGES variables, adjusted TRIAGES, and the adjusted four-original-variable model. These models addressed distinct analytical questions: The multivariable TRIAGES models were used to evaluate the conditional association and prognostic performance of TRIAGES after accounting for selected clinical and radiological severity indicators, whereas the TRIAGES-alone model evaluated standalone prognostic performance and its comparison with admission GCS quantified incremental discrimination relative to GCS alone. The adjusted models were not interpreted as demonstrating independent biological information provided by TRIAGES. As an additional reviewer-requested analysis, we constructed a clinically enriched GCS model including admission GCS, sex, injury mechanism, number of non-reactive pupils, and Rotterdam CT score and compared its discrimination with the otherwise analogous adjusted TRIAGES model in which TRIAGES replaced admission GCS. Because GCS is a component of TRIAGES, TRIAGES and admission GCS were not entered simultaneously in this comparison. AUC differences were assessed using paired DeLong tests, with corresponding 95% confidence intervals calculated from the DeLong covariance estimates. AUC 95% confidence intervals for individual models were estimated using 500 non-parametric bootstrap resamples. Nested models were compared using likelihood-ratio tests, and model fit was summarized using the Akaike information criterion. Unless otherwise specified, discrimination, calibration, Brier score, and DCA estimates for the comparative models were apparent performance estimates obtained from the same analytic dataset used for model fitting and were not optimism-corrected.

Internal validation of the TRIAGES-alone model was performed using 1,000 non-parametric bootstrap resamples of the single-imputation analytic cohort. In each resample, the model was refitted and performance was evaluated in both the bootstrap sample and the original cohort. Mean signed optimism was subtracted from apparent performance to obtain optimism-corrected AUC, Brier score, calibration intercept, and calibration slope. This bootstrap procedure was used to validate the single-imputation TRIAGES-alone model and did not constitute bootstrap validation of the pooled multiple-imputation association analysis.

Sensitivity analyses examined alternative missing-data strategies, alternative outcome definitions, retention of the single respiratory-rate value of 0, exclusion of patients recorded as intubated at admission, and restriction to isolated TBI. Prespecified subgroup analyses were performed by sex, age (<65 vs. > = 65 years) (11), and admission GCS severity (mild, 13–15; moderate, 9–12; severe, 3–8) (12). Interaction was assessed using TRIAGES-by-subgroup interaction terms. Subgroup analyses were exploratory and were not adjusted for multiple comparisons. All tests were two-sided, and a *p*-value of < 0.05 was considered statistically significant. To evaluate potential selection associated with unavailable discharge GOS, observed baseline characteristics were compared between the 753 included patients and the 57 patients excluded because of unavailable or invalid discharge GOS, with standardized mean differences additionally reported. As a bounding sensitivity analysis, the 57 excluded patients were alternatively assigned a favorable functional outcome or an unfavorable functional outcome, and the primary adjusted association was re-estimated using the same multiple-imputation and Model 2 framework (Supplementary Materials S1).

Statistical analyses were performed using Python version 3.13.5 with pandas, NumPy, SciPy, statsmodels, patsy, and scikit-learn. Figures were generated using Matplotlib, and multiple imputation was implemented according to the prespecified analytical procedure.

### 2.6 Ethics statement

This retrospective study was reviewed and approved by the Ethics Committee of Shishi Municipal Hospital (approval No. 2026060). The study was conducted in accordance with the principles of the Declaration of Helsinki and relevant institutional requirements. Given the retrospective study, which involved the secondary analysis of existing clinical data, the requirement for written informed consent was waived by the Ethics Committee.

## 3 Results

### 3.1 Study population and baseline characteristics

Figure 1 summarizes cohort assembly. Among 998 hospitalization records screened, duplicate reconciliation yielded 953 unique hospitalization records. After exclusion of 143 patients aged ≤18 years or with unavailable age, 810 adult patients remained. Of these, 57 were excluded because discharge GOS was unavailable or invalid, leaving 753 patients in the primary analytic cohort. Review of the original records confirmed that the 57 excluded patients had either been transferred to another hospital or discharged at the request of the patient/family after declining further treatment at our institution. Compared with included patients, the excluded patients were older (59.2 ± 14.5 vs. 53.7 ± 16.6 years; *p* = 0.008; SMD = 0.355), although comparisons of several other baseline prognostic variables were limited by substantial missingness in the excluded group (Supplementary Materials S1).

**Figure 1:**
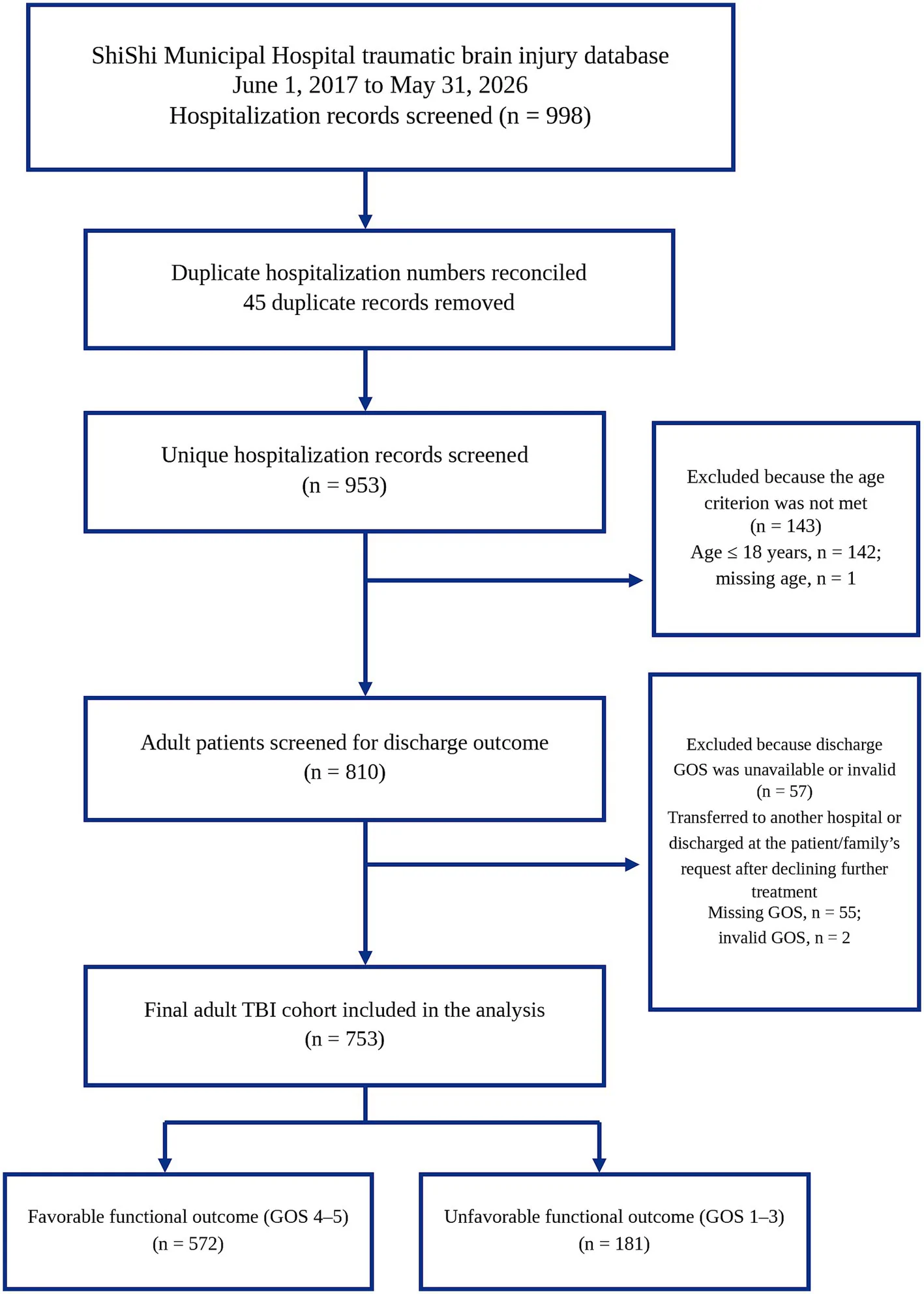
Study cohort selection and outcome classification. Flow diagram of cohort assembly from 998 TBI hospitalization records. After reconciliation of duplicate hospitalization identifiers, 953 unique hospitalization records were screened. Following exclusion of 143 patients who did not meet the age criterion (age ≤ 18 years, *n* = 142; missing age, *n* = 1), 810 adult patients were screened for discharge outcome. Of these, 57 were excluded because discharge GOS was unavailable or invalid, reflecting transfer to another hospital or discharge at the patient/family’s request after declining further treatment (missing GOS, *n* = 55; invalid GOS, *n* = 2). The final analytic cohort comprised 753 adult patients, including 572 with a favorable functional outcome (GOS 4–5) and 181 with an unfavorable functional outcome (GOS 1–3). GOS, Glasgow Outcome Scale; TBI, traumatic brain injury.

Baseline characteristics are summarized in Table 1. Compared with patients with favorable discharge GOS, those with unfavorable discharge GOS were older (58.6 ± 17.6 vs. 52.1 ± 16.0 years, *p* < 0.001), had higher TRIAGES scores (median 5 [IQR 4–6] vs. 1 [IQR 1–3], *p* < 0.001), and had lower admission GCS scores (median 6 [IQR 4–10] vs. 14 [IQR 12–15], *p* < 0.001). Unfavorable functional outcome was also associated with a greater frequency of impaired pupillary reactivity, a higher number of non-reactive pupils, and higher Rotterdam CT scores (all *p* < 0.001). White blood cell count, absolute neutrophil and monocyte counts, blood glucose, serum potassium, and fibrinogen also differed between outcome groups, whereas sex and injury mechanism did not differ significantly. TRIAGES scores ranged from 0 to 10 in the analytic cohort; although the theoretical scale ranges from 0 to 14, scores of 11–14 were not observed (Supplementary Materials S2).

**Table 1:** Baseline characteristics of the study population according to discharge GOS status.

Characteristic	Overall (*n* = 753)	Favorable discharge GOS (GOS 4–5; *n* = 572)	Unfavorable discharge GOS (GOS 1–3; *n* = 181)	*p*-value	SMD
Age, years	53.7 ± 16.6	52.1 ± 16.0	58.6 ± 17.6	<0.001	0.384
Sex, *n* (%)				0.147	0.126
Male	512 (68.0)	381 (66.6)	131 (72.4)		
Female	241 (32.0)	191 (33.4)	50 (27.6)		
TRIAGES score	2 [1–4]	1 [1–3]	5 [4–6]	<0.001	1.960
Admission GCS score	14 [9–15]	14 [12–15]	6 [4–10]	<0.001	1.809
Respiratory rate, breaths/min	20 [20–21]	20 [20–21]	20 [20–21]	0.274	0.041
Heart rate, beats/min	81 [74–90]	80 [73–89]	85 [76–98]	<0.001	0.398
Systolic blood pressure, mmHg	134 [123–156]	132 [122–150]	146 [125–169]	<0.001	0.459
Diastolic blood pressure, mmHg	80 [73–89]	80 [72–87]	80 [74–93]	0.039	0.235
Body temperature, °C	36.6 [36.5–36.8]	36.6 [36.5–36.8]	36.6 [36.5–36.9]	0.403	0.167
Left pupillary reactivity, *n* (%)				<0.001	0.996
Brisk	558 (80.8)	477 (90.2)	81 (50.0)		
Sluggish	56 (8.1)	30 (5.7)	26 (16.0)		
Absent/non-reactive	77 (11.1)	22 (4.2)	55 (34.0)		
Right pupillary reactivity, *n* (%)				<0.001	1.019
Brisk	573 (80.7)	490 (90.1)	83 (50.0)		
Sluggish	51 (7.2)	32 (5.9)	19 (11.4)		
Absent/non-reactive	86 (12.1)	22 (4.0)	64 (38.6)		
Number of non-reactive pupils, *n* (%)				<0.001	0.953
0	592 (85.7)	498 (94.1)	94 (58.0)		
1	35 (5.1)	18 (3.4)	17 (10.5)		
2	64 (9.3)	13 (2.5)	51 (31.5)		
Rotterdam CT score	2 [2–3]	2 [1–3]	4 [3–5]	<0.001	1.200
Injury mechanism, *n* (%)				0.088	0.219
Traffic accident	83 (11.5)	56 (10.1)	27 (16.1)		
Fall from height	48 (6.7)	34 (6.2)	14 (8.3)		
Ground-level fall	93 (12.9)	76 (13.8)	17 (10.1)		
Other	496 (68.9)	386 (69.9)	110 (65.5)		
White blood cell count, ×10^9^/L	13.45 [10.08–17.41]	12.78 [9.77–16.30]	15.47 [11.00–20.11]	<0.001	0.393
Absolute neutrophil count, ×10^9^/L	10.64 [7.20–14.90]	10.21 [7.03–14.19]	12.77 [7.95–17.13]	<0.001	0.320
Absolute lymphocyte count, ×10^9^/L	1.50 [0.98–2.40]	1.49 [0.99–2.30]	1.70 [0.90–2.99]	0.130	0.245
Absolute monocyte count, ×10^9^/L	0.71 [0.50–1.01]	0.70 [0.50–0.95]	0.90 [0.61–1.29]	<0.001	0.447
RDW-CV, %	12.9 [12.4–13.5]	12.8 [12.4–13.4]	13.0 [12.5–13.7]	0.016	0.135
Platelet count, ×10^9^/L	225.8 ± 66.1	227.9 ± 67.1	219.1 ± 62.3	0.116	0.134
Blood glucose, mmol/L	7.59 [6.44–9.28]	7.26 [6.28–8.54]	9.12 [7.72–10.96]	<0.001	0.591
Serum potassium, mmol/L	3.59 ± 0.48	3.62 ± 0.46	3.49 ± 0.53	0.005	0.257
Fibrinogen, g/L	2.32 [1.85–2.98]	2.39 [1.93–3.04]	2.08 [1.64–2.83]	<0.001	0.274

Data are presented as mean ± standard deviation, median [interquartile range], or *n* (%). Percentages were calculated using non-missing observations for each variable. *p*-values compare the favorable and unfavorable discharge GOS groups and were calculated using Welch’s *t*-test, the Mann–Whitney *U*-test, or Pearson’s chi-squared test, as appropriate. SMDs are absolute standardized mean differences; generalized SMDs are reported for multicategory variables. Continuous-variable SMDs were calculated from group means and pooled standard deviations, irrespective of the descriptive summary shown.

CT, computed tomography; GCS, Glasgow Coma Scale; GOS, Glasgow Outcome Scale; IQR, interquartile range; RDW-CV, red blood cell distribution width-coefficient of variation; SMD, standardized mean difference; TRIAGES, Trauma Rating Index in Age, Glasgow Coma Scale, Respiratory rate, and Systolic blood pressure. Detailed variable-level missingness is reported in Supplementary Material S1.

### 3.2 Association between TRIAGES score and unfavorable functional status

In the unadjusted logistic regression model, each 1-point increase in the TRIAGES score was associated with higher odds of unfavorable functional status at discharge (OR 3.13, 95% CI 2.63–3.72; *p* < 0.001) (Table 2). After adjustment for sex, injury mechanism, number of non-reactive pupils, and Rotterdam CT score modeled with an RCS (Model 2, the prespecified primary adjusted model), the association remained significant (adjusted OR 2.81, 95% CI 2.29–3.45; *p* < 0.001). Further adjustment for blood glucose, white blood cell count, platelet count, and fibrinogen produced a similar estimate (Model 3: adjusted OR 2.85, 95% CI 2.31–3.52; *p* < 0.001). Per 1-standard-deviation increase in TRIAGES (approximately 1.95 points), the adjusted OR was 7.50 (95% CI 5.03–11.19) in Model 2.

**Table 2:** Association between the TRIAGES score and unfavorable functional status at hospital discharge.

Model	Adjustment	OR per 1-point increase (95% CI)	*p*-value	OR per 1-SD increase (95% CI)	*p*-value
Model 1	Unadjusted	3.13 (2.63–3.72)	<0.001	9.27 (6.61–12.98)	<0.001
Model 2	Sex, injury mechanism, number of non-reactive pupils, and Rotterdam CT score (RCS; primary model)	2.81 (2.29–3.45)	<0.001	7.50 (5.03–11.19)	<0.001
Model 3	Model 2 plus blood glucose, white blood cell count, platelet count, and fibrinogen	2.85 (2.31–3.52)	<0.001	7.70 (5.10–11.62)	<0.001

Odds ratios were estimated using logistic regression models fitted separately in 20 multiply imputed datasets and pooled using Rubin’s rules. The primary outcome was unfavorable functional status at hospital discharge, defined as a Glasgow Outcome Scale score of 1–3. The TRIAGES score was modeled continuously; one standard deviation corresponded to approximately 1.95 points. Restricted cubic spline assessment supported a linear functional form for TRIAGES (P for non-linearity = 0.366). Rotterdam CT score showed evidence of non-linearity and was therefore modeled using a three-knot restricted cubic spline with knots at scores 1, 2, and 5 in Models 2 and 3. Model 2 was the prespecified primary adjusted model.

CI, confidence interval; CT, computed tomography; GOS, Glasgow Outcome Scale; OR, odds ratio; RCS, restricted cubic spline; SD, standard deviation; TRIAGES, Trauma Rating Index in Age, Glasgow Coma Scale, Respiratory rate, and Systolic blood pressure.

### 3.3 Dose–response relationship

RCS analysis showed a strong overall association between TRIAGES and unfavorable discharge GOS in the primary adjusted model (P overall < 0.001), with no evidence of departure from linearity (P for non-linearity = 0.366) (Table 3; Figure 2). The corresponding tests for non-linearity were also non-significant in the unadjusted model (*p* = 0.259) and the extended adjusted model (*p* = 0.377). Accordingly, the linear effect estimate per 1-point increase in TRIAGES was retained as the principal measure of association.

**Table 3:** Restricted cubic spline analysis of the association between the TRIAGES score and unfavorable functional status at hospital discharge.

Model	Adjustment	Linear OR per 1-point increase (95% CI)	P overall	P for non-linearity	Final functional form
Model 1	Unadjusted	3.13 (2.63–3.72)	<0.001	0.259	Linear
Model 2	Sex, injury mechanism, number of non-reactive pupils, and Rotterdam CT score	2.81 (2.29–3.45)	<0.001	0.366	Linear
Model 3	Model 2 plus blood glucose, white blood cell count, platelet count, and fibrinogen	2.85 (2.31–3.52)	<0.001	0.377	Linear

Data are odds ratios with 95% confidence intervals unless otherwise indicated. Restricted cubic spline specification: three knots were placed at the 10th, 50th, and 90th percentiles of the TRIAGES score distribution (scores 0, 2, and 5, respectively), with a TRIAGES score of 2 as the reference. P overall was obtained from a pooled 2-degree-of-freedom Wald test of the linear and non-linear spline terms; P for non-linearity tested the non-linear spline component. Because no model showed evidence of non-linearity, the linear odds ratio per 1-point increase was retained as the principal effect estimate. Model 2 was the primary adjusted model and was used for Figure 2. Rotterdam CT score was modeled using a three-knot restricted cubic spline in Models 2 and 3. Models were fitted in each of 20 multiply imputed datasets, and estimates were combined using Rubin’s rules.

CI, confidence interval; CT, computed tomography; GOS, Glasgow Outcome Scale; OR, odds ratio; TRIAGES, Trauma Rating Index in Age, Glasgow Coma Scale, Respiratory rate, and Systolic blood pressure.

**Figure 2:**
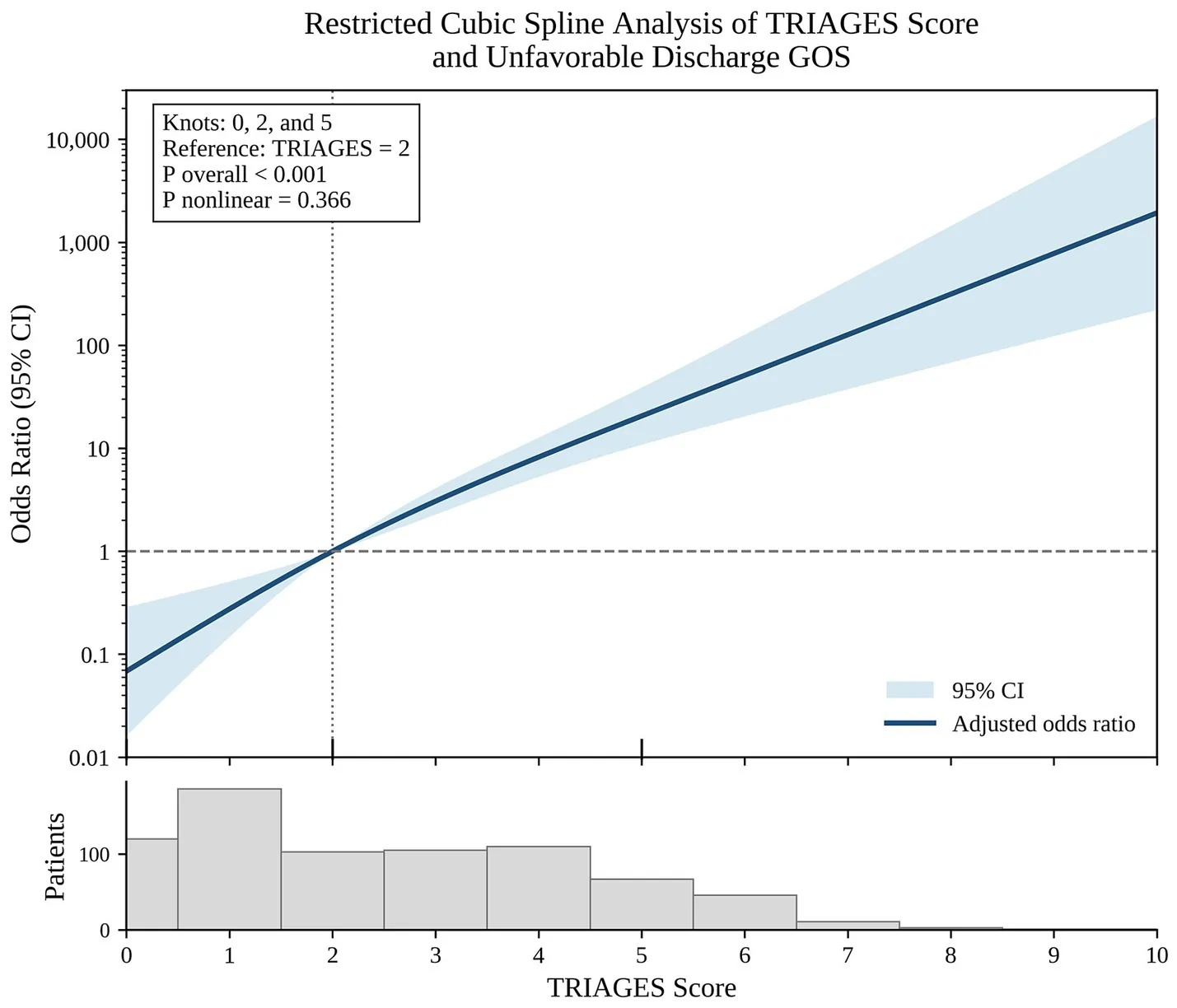
RCS association between TRIAGES score and unfavorable functional status at discharge. The solid line shows the adjusted odds ratio and the shaded area the 95% confidence interval from the primary adjusted model. Three knots were placed at TRIAGES scores 0, 2, and 5, corresponding to the 10th, 50th, and 90th percentiles, with TRIAGES = 2 as the reference. The model was adjusted for sex, injury mechanism, number of non-reactive pupils, and Rotterdam CT score; Rotterdam CT score was modeled using a three-knot restricted cubic spline. The overall association was significant (*p* < 0.001), with no evidence of non-linearity (*p* = 0.366). The lower panel shows the distribution of TRIAGES scores in the analytic cohort. CI, confidence interval; CT, computed tomography; TRIAGES, Trauma Rating Index in Age, Glasgow Coma Scale, Respiratory rate, and Systolic blood pressure.

### 3.4 Prognostic performance of the TRIAGES score

The TRIAGES-alone model demonstrated good discrimination for unfavorable functional status at discharge, with an AUC of 0.905 (Figure 3A). Within the study cohort, the Youden index identified TRIAGES ≥3 as the data-derived operating threshold, corresponding to a sensitivity of 0.956 and a specificity of 0.701. The apparent Brier score was 0.101. The calibration curve showed close agreement between predicted and observed probabilities (Figure 3B), and DCA showed positive net benefit across the clinically evaluated threshold-probability range compared with the treat-none strategy and, over most of the range, the treat-all strategy (Figure 3C).

**Figure 3:**
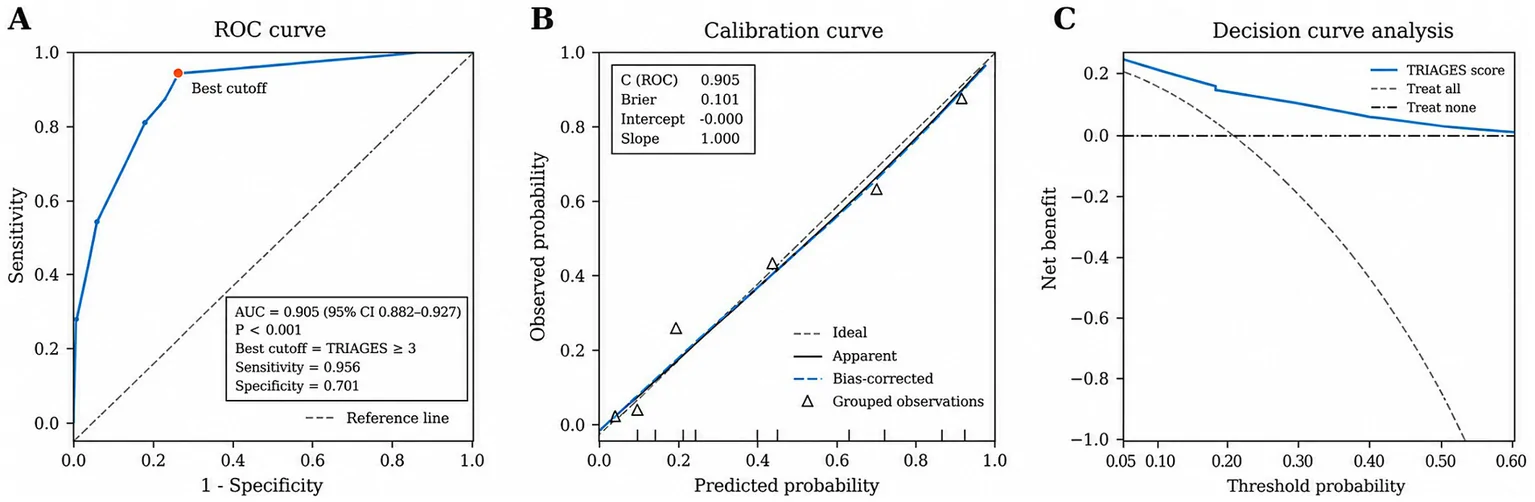
Discrimination, calibration, and clinical utility of the TRIAGES-alone model. **(A)** ROC curve for the TRIAGES score as the sole predictor of unfavorable discharge GOS. The AUC was 0.905; the cohort-derived Youden threshold was TRIAGES ≥3, with a sensitivity of 0.956 and specificity of 0.701. **(B)** Apparent and bootstrap bias-corrected calibration curves, with grouped observed probabilities and the 45° ideal line. The apparent Brier score was 0.101. **(C)** Decision curve analysis showing the net benefit of the TRIAGES-alone model across threshold probabilities from 0.05 to 0.60, together with treat-all and treat-none strategies. AUC, area under the ROC curve; GOS, Glasgow Outcome Scale; TRIAGES, Trauma Rating Index in Age, Glasgow Coma Scale, Respiratory rate, and Systolic blood pressure.

### 3.5 Apparent comparative and incremental prognostic performance

Apparent comparative performance of the five prespecified models is shown in Table 4 and Figure 4. The AUC was 0.890 for admission GCS alone, 0.905 for TRIAGES alone, 0.906 for the four original TRIAGES variables, 0.922 for the adjusted TRIAGES model, and 0.920 for the adjusted four-original-variable model. Within the present cohort, TRIAGES alone showed a small increase in discrimination relative to admission GCS alone (AUC 0.905 vs. 0.890; ΔAUC 0.015, 95% CI 0.001–0.029; paired DeLong *p* = 0.034). Among the apparent estimates, the adjusted TRIAGES model had the highest AUC and the lowest AIC (464.5), with a Brier score of 0.095. The ROC, calibration, and decision-curve panels showed broadly concordant apparent performance patterns across the five models (Figure 4). In the additional clinically enriched comparison, the model containing admission GCS, sex, injury mechanism, number of non-reactive pupils, and Rotterdam CT score had an AUC of 0.902. Replacing admission GCS with TRIAGES while retaining the same additional covariates yielded an AUC of 0.922 (ΔAUC 0.020, 95% CI 0.009–0.032; paired DeLong *p* < 0.001). These comparative estimates were obtained in the same dataset used for model fitting and were not optimism-corrected.

**Table 4:** Apparent comparative performance of five prediction models for unfavorable functional outcome at hospital discharge.

Model	AUC (95% CI)	ΔAUC vs. GCS (95% CI)	DeLong *p*-value	Likelihood-ratio test *p*-value	AIC	Brier score
Admission GCS alone	0.890 (0.863–0.913)	Reference	Reference	Reference	537.5	0.112
TRIAGES alone	0.905 (0.884–0.926)	0.015 (0.001–0.029)	0.034	NA	480.2	0.101
Four original variables	0.906 (0.881–0.929)	0.016 (−0.000–0.032)	0.054	<0.001	492.5	0.100
Adjusted TRIAGES	0.922 (0.903–0.940)	0.032 (0.018–0.046)	<0.001	<0.001	464.5	0.095
Adjusted four original variables	0.920 (0.900–0.939)	0.030 (0.012–0.048)	0.001	<0.001	480.2	0.095

Data are presented as apparent performance estimates from the 753-patient analytic dataset used to generate the corresponding ROC, calibration, and decision-curve figure. Model definitions: GCS alone included admission GCS; TRIAGES alone included the TRIAGES score; the four-original-variable model included age, admission GCS, respiratory rate, and systolic blood pressure. The adjusted TRIAGES model additionally included sex, injury mechanism, number of non-reactive pupils, and Rotterdam CT score. The adjusted four-original-variable model included the same adjustment variables plus the four original TRIAGES components. Rotterdam CT score was modeled using a three-knot restricted cubic spline in the adjusted models. AUC 95% confidence intervals for individual models were estimated using 500 non-parametric bootstrap resamples. ΔAUC 95% confidence intervals and paired DeLong *p*-values use the GCS-alone model as the reference and were calculated from the paired DeLong covariance estimates. As an additional reviewer-requested analysis, a clinically enriched GCS model including admission GCS, sex, injury mechanism, number of non-reactive pupils, and Rotterdam CT score had an AUC of 0.902; the otherwise analogous adjusted TRIAGES model had an AUC of 0.922 (ΔAUC = 0.020, 95% CI 0.009–0.032; paired DeLong *p* < 0.001). Because GCS is a component of TRIAGES, GCS and TRIAGES were not entered simultaneously in this comparison. Likelihood-ratio tests were applied only to nested model comparisons. Lower AIC and Brier scores indicate better model fit and overall prediction accuracy, respectively. Apparent calibration intercepts and slopes equal 0 and 1 by construction because the models were evaluated in the same dataset used for fitting. Clinical utility was assessed using decision curve analysis.

AIC, Akaike’s information criterion; AUC, area under the ROC curve; CI, confidence interval; CT, computed tomography; GCS, Glasgow Coma Scale; GOS, Glasgow Outcome Scale; NA, not applicable; TRIAGES, Trauma Rating Index in Age, Glasgow Coma Scale, Respiratory rate, and Systolic blood pressure.

**Figure 4:**
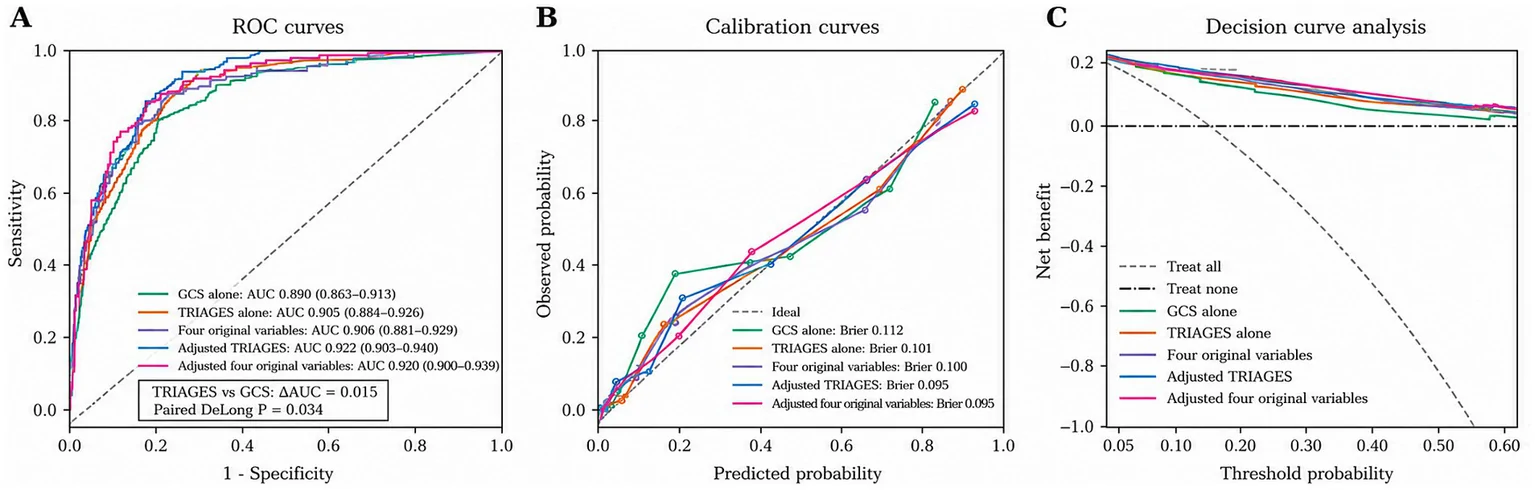
Apparent comparative predictive performance of five models for unfavorable functional outcome at discharge. **(A)** ROC curves for admission GCS alone, TRIAGES alone, the four original TRIAGES variables, the adjusted TRIAGES model, and the adjusted four-original-variable model. TRIAGES alone had an AUC of 0.905 compared with 0.890 for GCS alone (ΔAUC = 0.015; paired DeLong *p* = 0.034). **(B)** Calibration curves for the five models, with Brier scores shown in the legend and the 45° ideal line for reference. **(C)** Decision curve analysis comparing net benefit across threshold probabilities. Adjusted models included sex, injury mechanism, number of non-reactive pupils, and Rotterdam CT score; Rotterdam CT score was modeled with a restricted cubic spline. AUC, area under the ROC curve; GCS, Glasgow Coma Scale; CT, computed tomography; TRIAGES, Trauma Rating Index in Age, Glasgow Coma Scale, Respiratory rate, and Systolic blood pressure.

### 3.6 Bootstrap internal validation of the TRIAGES-alone model

Internal validation of the TRIAGES-alone model was performed using 1,000 non-parametric bootstrap resamples of the single-imputation analytic cohort (*n* = 753; 181 unfavorable functional outcome). The apparent AUC was 0.905, and the mean signed optimism was 0.000, yielding an optimism-corrected AUC of 0.905. The optimism-corrected Brier score was 0.102, while the corrected calibration intercept and slope were 0.008 and 0.998, respectively (Figure 5; Supplementary Table S5). These estimates indicated minimal optimism in model performance within the analytic cohort.

**Figure 5:**
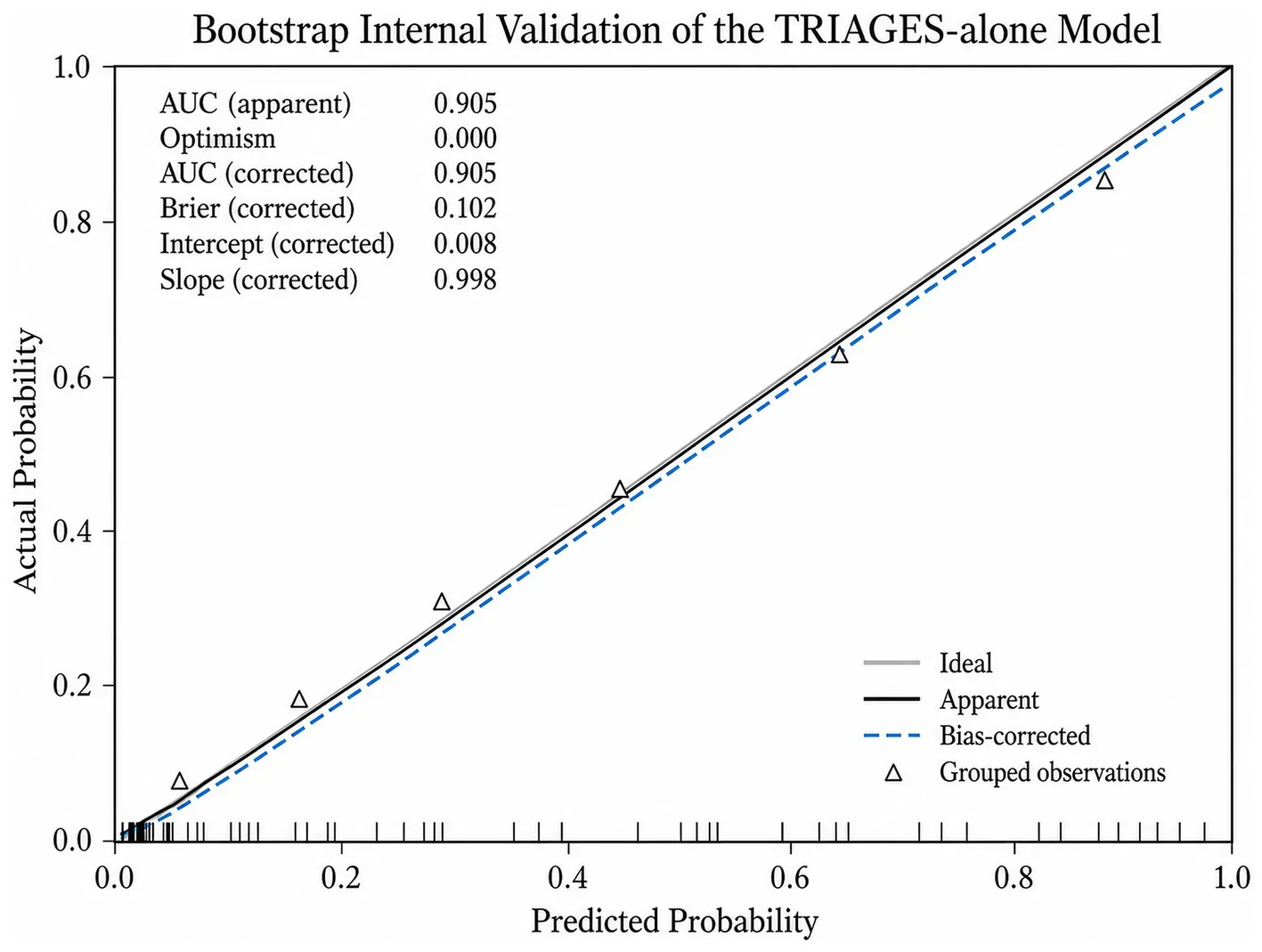
Bootstrap internal validation of the TRIAGES-alone model. Calibration plot based on 1,000 non-parametric bootstrap resamples of the single-imputation analytic cohort. The apparent AUC was 0.905, and the optimism-corrected AUC was 0.905. The corrected Brier score was 0.102, the corrected calibration intercept was 0.008, and the corrected calibration slope was 0.998. The gray line denotes ideal calibration, the black line the apparent calibration, the dashed line the bias-corrected calibration, and triangles grouped observed probabilities; rug marks show the distribution of predicted probabilities. AUC, area under the ROC curve.

### 3.7 Sensitivity and subgroup analyses

The association between TRIAGES and unfavorable functional outcome remained statistically significant across all prespecified sensitivity analyses (Figure 6A; Supplementary Material S4). The primary MICE estimate was 2.81 (95% CI 2.29–3.45) per 1-point increase. Comparable estimates were obtained in the complete-case analysis (OR 2.76, 95% CI 2.24–3.40) and single-imputation analysis (OR 2.86, 95% CI 2.33–3.50). The association was also maintained when mRS 3–6 was used as an alternative binary outcome (OR 2.54, 95% CI 2.10–3.07), when GOS 1–5 was modeled ordinally (common OR 1.97, 95% CI 1.75–2.22), after exclusion of in-hospital deaths (OR 3.06, 95% CI 2.41–3.90), when the respiratory-rate value of 0 was retained as observed (OR 2.81, 95% CI 2.29–3.45), after exclusion of patients intubated at admission (OR 2.78, 95% CI 2.26–3.41), and in patients with isolated TBI (OR 2.99, 95% CI 2.33–3.84). This association was also present in sensitivity analyses using extreme-case scenarios for missing discharge outcomes. If all 57 excluded patients had a good functional outcome, the adjusted OR was 2.75 (95% CI 2.25–3.36; *p* < 0.001); if all 57 patients had a poor functional outcome, the corresponding OR was 2.33 (95% CI 1.97–2.75; *p* < 0.001) (Supplementary Materials S1).

**Figure 6:**
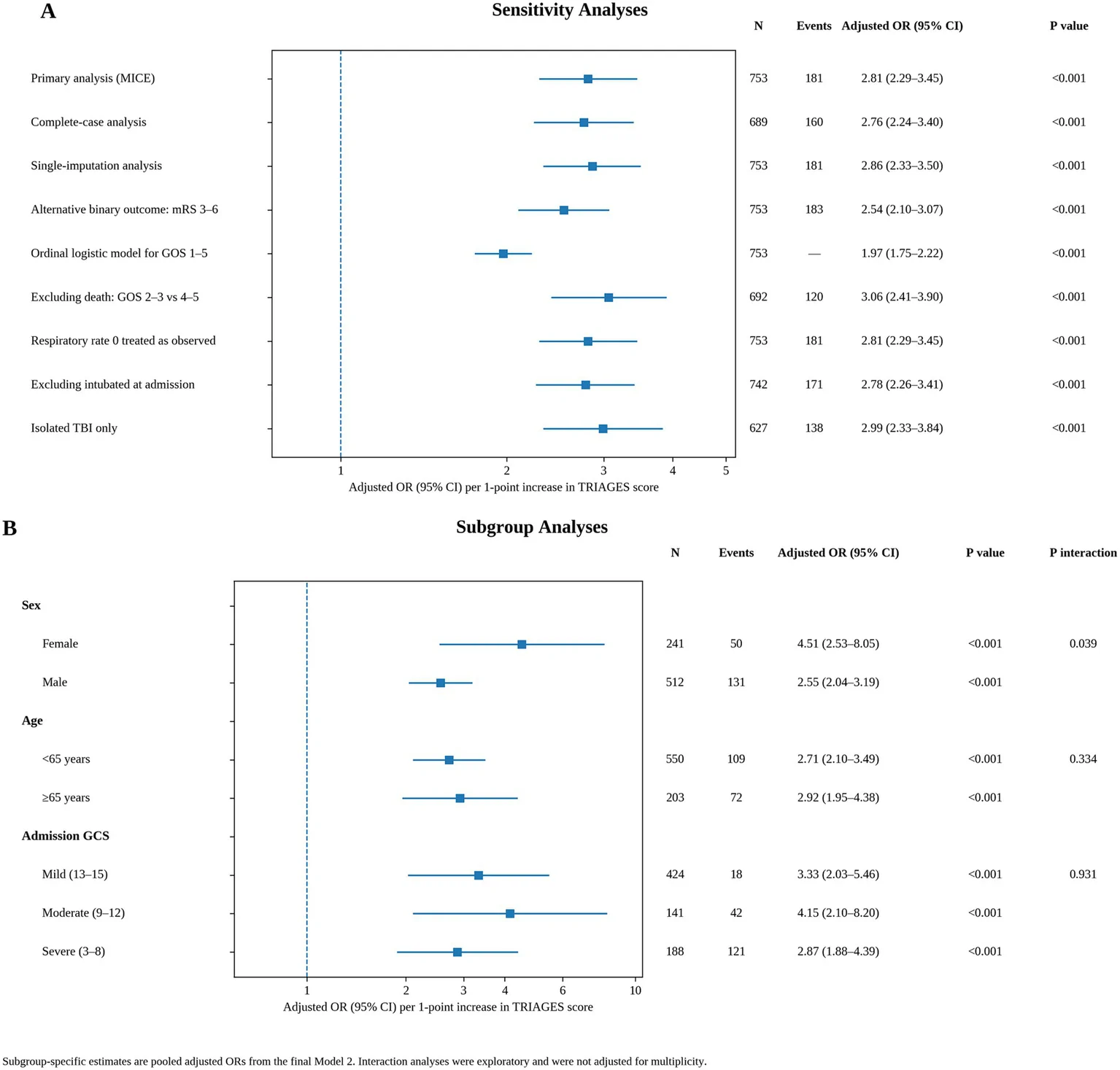
Sensitivity and subgroup analyses of the association between TRIAGES score and discharge outcomes. **(A)** Sensitivity analyses reporting adjusted odds ratios per 1-point increase in TRIAGES under alternative missing-data approaches, outcome definitions, and restricted analytic populations. **(B)** Subgroup analyses stratified by sex, age, and admission GCS severity, with corresponding interaction *p*-values. Estimates were based on the final primary Model 2 framework, which adjusted for sex, injury mechanism, number of non-reactive pupils, and Rotterdam CT score modeled with a three-knot restricted cubic spline; the stratifying variable was omitted from the adjustment set when appropriate. Subgroup analyses were exploratory and were not adjusted for multiplicity. CI, confidence interval; GCS, Glasgow Coma Scale; GOS, Glasgow Outcome Scale; MICE, multiple imputation by chained equations; mRS, modified Rankin Scale; OR, odds ratio; TBI, traumatic brain injury; TRIAGES, Trauma Rating Index in Age, Glasgow Coma Scale, Respiratory rate, and Systolic blood pressure.

In subgroup analyses (Figure 6B), higher TRIAGES scores were associated with unfavorable discharge GOS in both women (OR 4.51, 95% CI 2.53–8.05) and men (OR 2.55, 95% CI 2.04–3.19), with evidence of interaction by sex (P for interaction = 0.039). No significant interaction was detected by age (<65 vs. ≥65 years; P for interaction = 0.334) or admission GCS severity (mild, moderate, or severe TBI; P for interaction = 0.931). Subgroup analyses were exploratory and were not adjusted for multiple comparisons.

## 4 Discussion

In this retrospective cohort of adults with TBI, a higher TRIAGES score assessed at initial emergency department presentation was independently associated with an increased likelihood of unfavorable functional status at hospital discharge. The association remained robust after adjustment for relevant clinical and radiological factors and showed no clear evidence of non-linearity, supporting a broadly graded relationship between increasing TRIAGES scores and worse functional outcome. Beyond its association with outcome, the TRIAGES-alone model demonstrated good prognostic performance, including discrimination, calibration, and clinical net benefit, and showed minimal optimism on bootstrap internal validation. Comparative performance of the competing models was assessed using apparent within-cohort estimates and should therefore be interpreted cautiously. The association findings were generally consistent across multiple sensitivity analyses. These observations are in line with contemporary TBI studies showing that early clinical characteristics contain substantial prognostic information for functional outcomes and that recovery after TBI is heterogeneous across patients and over time (13–16).

The prognostic relevance of TRIAGES is clinically plausible because its four components capture complementary dimensions of early injury severity. Increasing age is a well-established predictor of mortality and unfavorable functional outcome after TBI, potentially reflecting reduced physiological reserve and recovery capacity (17). GCS provides a direct bedside assessment of neurological dysfunction, and lower GCS components, particularly the motor response, are strongly associated with subsequent functional outcome (18). Respiratory rate is a non-specific but immediately available indicator of respiratory and systemic physiological instability; together with GCS and SBP, it has long contributed to physiological trauma severity assessment (19). SBP additionally reflects circulatory perfusion, and hypotension after severe TBI is consistently associated with poorer outcome, supporting current recommendations to avoid inadequate blood pressure and secondary cerebral hypoperfusion (20, 21). Thus, TRIAGES integrates age-related vulnerability, neurological impairment, respiratory physiology, and circulatory status into a single bedside score, which may explain its association with discharge functional status.

These findings are broadly consistent with previous studies showing that TRIAGES has prognostic value in patients with TBI. In 2023, Jiang et al. evaluated 1,156 patients with isolated acute TBI and reported that TRIAGES showed good discrimination for 24-h in-hospital mortality, with performance comparable to that of GCS and the Revised Trauma Score (6). In 2024, Jiang et al. further examined 982 patients aged ≥65 years with isolated TBI and found that TRIAGES remained useful for predicting 24-h mortality and showed slightly higher discrimination than GCS (7). The present study extends these observations in several respects. First, this study evaluated unfavorable functional status at hospital discharge rather than very early mortality alone. This endpoint incorporates not only death but also severe neurological disability and therefore provides a broader assessment of the early functional burden after brain injury (22, 23). Second, the cohort encompassed adults across different age groups and admission GCS severity categories, while the association remained consistent when the analysis was restricted to isolated TBI. Previous study has shown substantial variation in acute functional outcome across age and TBI severity groups (24). Third, in addition to multivariable association and discrimination, the study assessed dose–response relationships, calibration, Brier score, clinical net benefit, bootstrap internal validation, and multiple sensitivity and subgroup analyses, providing a more comprehensive evaluation of the prognostic utility and robustness of TRIAGES.

The analyses in the present study address several related but distinct questions. The adjusted association between TRIAGES and unfavorable functional outcome represents a conditional statistical association after accounting for selected clinical and radiological severity indicators and should not be interpreted as evidence of independent biological information. Rather, TRIAGES may primarily function as a compact index of early clinical severity. Within this cohort, TRIAGES showed a statistically significant but small increase in discrimination compared with admission GCS alone (AUC 0.905 vs. 0.890; ΔAUC = 0.015, 95% CI 0.001–0.029; paired DeLong *p* = 0.034), and the clinical importance of this difference remains uncertain. In the clinically enriched comparison, the admission GCS-based model had an AUC of 0.902, whereas the otherwise analogous TRIAGES-based model had an AUC of 0.922 (ΔAUC = 0.020, 95% CI 0.009–0.032; paired DeLong *p* < 0.001). This suggests that TRIAGES may provide complementary prognostic information beyond GCS within this cohort, but not biological independence or external superiority. Previous TBI studies have similarly shown that combining GCS with additional early clinical characteristics can improve prognostic information while preserving bedside simplicity (25–27). The four original TRIAGES variables showed virtually identical discrimination to the point-based score, suggesting that TRIAGES retained most of their discriminatory information while offering greater simplicity. Nevertheless, integrated point scores may sacrifice some prognostic information compared with separately modeled predictors (28). Therefore, the principal advantage of TRIAGES may lie in balancing prognostic information with clinical simplicity rather than in markedly outperforming GCS.

From a clinical perspective, TRIAGES may be most useful as an early risk-stratification tool rather than as a stand-alone prognostic instrument. Because the score relies only on variables routinely available at emergency presentation, it could help identify patients who may warrant closer neurological observation, earlier neurosurgical or critical care assessment, and more structured risk communication, including in settings where advanced diagnostic resources are limited (29). Within the present cohort, the Youden index-derived TRIAGES threshold of ≥3 showed high sensitivity but only moderate specificity. However, because this threshold was selected and evaluated in the same dataset, it should be regarded as an exploratory, cohort-specific operating point rather than a clinically validated decision threshold. Its transportability and clinical usefulness require assessment in independent populations before any threshold-based application can be considered. Importantly, prognostic scores should complement, rather than replace, comprehensive clinical assessment. Contemporary neuroprognostication guidance emphasizes substantial uncertainty even when validated predictors or models are used, particularly when prognosis informs high-stakes decisions (30). TRIAGES, therefore, should not be used in isolation to determine surgery, ICU admission, treatment limitation, or withdrawal of life-sustaining therapy because premature prognostic certainty may contribute to self-fulfilling treatment decisions (31). Instead, it may serve as one component of multidisciplinary assessment and clinician–family communication, in which prognostic estimates are explicitly presented together with their uncertainty (32).

This study has a number of strengths. First, unlike previous investigations that focused on isolated TBI or elderly patients only, we included a large cohort of adults with TBI of all ages and admission GCS severities. Second, TRIAGES was constructed using the earliest physiologic and neurological measurements obtained after emergency department arrival and before any therapeutic intervention initiated at the hospital. Therefore, TRIAGES reflected the patient’s earliest in-hospital clinical presentation rather than a strictly treatment-naïve baseline state. Third, we addressed missing covariate data using multiple imputation and assessed the robustness of the primary association using multivariable adjustment and restricted cubic spline analysis. Fourth, prognostic performance was assessed using complementary measures including discrimination, calibration, Brier score, and DCA, with bootstrap internal validation specifically performed for the TRIAGES-alone model. Several limitations should also be acknowledged. First, selection and information bias, residual confounding, and lack of generalizability are inherent to retrospective single-center studies. Fifty-seven adults had to be excluded because discharge GOS was unavailable or invalid due to transfer to another hospital or discharge at the patient/family’s request following discontinuation of further treatment. These reasons for disposition may be related to prognosis or other unmeasured factors, introducing the possibility that missingness of outcome was informative and caused selection bias. Although best- and worst-case sensitivity analyses preserved the robustness of this association, residual selection bias cannot be ruled out. Second, the primary endpoint was GOS at hospital discharge rather than a standardized long-term outcome; differences in length of stay may influence discharge status, and discharge GOS cannot substitute for 3- or 6-month functional assessment. Third, the five-category GOS provides less granularity than the extended GOS. Fourth, although missing data were addressed using multiple imputation, the missing-at-random assumption cannot be empirically verified. Fifth, bootstrap internal validation was performed only for the TRIAGES-alone model. Comparative performance estimates for the other models were apparent estimates from the same dataset used for fitting and may therefore be optimistic. Moreover, internal bootstrap validation does not establish performance in independent populations. Sixth, while TRIAGES variables were collected prior to treatment beginning at our hospital, prehospital interventions/exposures that may have affected GCS, respiratory rate, or SBP (and thus the derived TRIAGES score) were not known. Such interventions/exposures include airway management, sedation or paralysis, fluid or vasoactive therapy, and alcohol/drug exposure. As such, TRIAGES should be considered an early, in-hospital evaluation rather than a strictly treatment-naïve measure. Finally, discharge GOS was obtained from routine clinical records rather than a standardized structured interview, which may have introduced interobserver variability or outcome misclassification.

## 5 Conclusion

In adults with TBI, a higher TRIAGES score at initial emergency department presentation was independently associated with unfavorable functional status at hospital discharge. TRIAGES demonstrated good prognostic performance, with a small within-cohort increase in discrimination relative to admission GCS alone. The clinical importance of this difference remains uncertain. Its potential clinical value may therefore lie in integrating age and readily available physiological information with GCS in a simple bedside score rather than in substantially outperforming GCS. External validation and prospective studies with standardized long-term functional outcomes are warranted before broader clinical implementation.

## Data Availability

The datasets presented in this article are not readily available because they contain clinical information subject to patient privacy and ethical and institutional restrictions. Access to de-identified data may be granted to qualified researchers upon reasonable request and subject to approval by the Ethics Committee of Shishi Municipal Hospital and the relevant institutional authority. Requests to access the datasets should be directed to RY, asi6554@163.com.
