# Supplementary material for: Admission TRIAGES score for unfavorable functional outcome after traumatic brain injury: association, prognostic performance, and comparison with the Glasgow coma scale
Source: Front Neurol. 2026 Oct 2;17:1963243. doi: 10.3389/fneur.2026.1963243 (PMC13671951; doi:10.3389/fneur.2026.1963243)
Supplement: Supplementary file 1 [file Supplementary_file_1.docx]

**Supplementary Materials S1: Additional analyses addressing potential selection bias due to unavailable discharge GOS**

**Supplementary Methods**

Cohort review and included-versus-excluded comparison. After duplicate reconciliation and application of the age criterion, 810 adult patients remained eligible for outcome assessment. A valid discharge Glasgow Outcome Scale (GOS) could not be assigned to 57 patients (55 with missing GOS and 2 with an invalid recorded GOS value of 0). Review of the original medical records confirmed that these patients had either been transferred to another hospital or discharged at the request of the patient/family after declining further treatment at our institution. Observed baseline characteristics were compared between the 753 included patients and these 57 outcome-excluded patients. Continuous variables were compared using Welch's t test for age and Mann-Whitney U tests for other continuous variables; categorical variables were compared using chi-square or Fisher's exact tests, as appropriate. Standardized mean differences (SMDs) were additionally reported. Comparisons were based on observed pre-imputation data, and the number of available observations is shown for each variable.

Extreme-case sensitivity analysis. To bound the potential influence of unavailable discharge outcomes, the 57 excluded patients were added back to the analytic cohort and were alternatively assigned a favorable functional outcome (best-case scenario) or an unfavorable functional outcome (worst-case scenario). Missing baseline predictors were handled using the same multiple-imputation-by-chained-equations framework as the primary analysis (20 imputed datasets; predictive mean matching with five nearest donors for continuous variables and multinomial logistic imputation for categorical variables). TRIAGES and the number of nonreactive pupils were re-derived after imputation. The primary adjusted Model 2 framework was retained, including sex, injury mechanism, number of nonreactive pupils, and Rotterdam CT score; Rotterdam CT score was modeled using the same three-knot restricted cubic spline as in the primary analysis.

**Supplementary Results**

Included-versus-excluded comparison. The 57 patients excluded because discharge GOS was unavailable or invalid were older than the 753 included patients (59.2 ± 14.5 vs 53.7 ± 16.6 years; P = 0.008; SMD = 0.355). Sex distribution was similar between groups. Comparisons of several other prognostic variables were limited by substantial missingness in the excluded group; for example, admission GCS and directly calculable TRIAGES scores were available for only 14 of the 57 excluded patients, and Rotterdam CT scores were available for only 6. These comparisons therefore should not be interpreted as demonstrating equivalence between groups (Supplementary Table S1-1).

Extreme-case sensitivity analysis. The primary adjusted association was reproduced in the uploaded analytic dataset (OR 2.81, 95% CI 2.29-3.45; P < 0.001). When all 57 excluded patients were assigned a favorable functional outcome, the adjusted OR was 2.75 (95% CI 2.25-3.36; P < 0.001). When all 57 were assigned an unfavorable functional outcome, the association was attenuated but remained directionally consistent and statistically significant (OR 2.33, 95% CI 1.97-2.75; P < 0.001) (Supplementary Table S1-2).

**Supplementary Table S1-1. Comparison of observed baseline characteristics between included patients and patients excluded because discharge GOS was unavailable or invalid**

| **Characteristic** | **Included patients** | **Available n** | **Excluded patients** | **Available n** | **P value** | **SMD** |
| --- | --- | --- | --- | --- | --- | --- |
| Age, years | 53.7 ± 16.6 | 753 | 59.2 ± 14.5 | 57 | 0.008 | 0.355 |
| **Sex, n (%)** |  | 753 |  | 56 | 0.766 | 0.041 |
| Male | 512 (68.0%) |  | 37 (66.1%) |  |  |  |
| Female | 241 (32.0%) |  | 19 (33.9%) |  |  |  |
| TRIAGES score | 2 [1-4] | 752 | 2 [1-3] | 14 | 0.324 | 0.312 |
| Temperature, °C | 36.6 [36.5-36.8] | 753 | 36.6 [36.5-36.8] | 35 | 0.138 | 0.285 |
| Respiratory rate, breaths/min | 20 [20-21] | 752 | 20 [20-21] | 35 | 0.262 | 0.149 |
| Heart rate, beats/min | 81 [74-90] | 753 | 79 [73-86] | 35 | 0.496 | 0.048 |
| Systolic blood pressure, mmHg | 134 [123-156] | 753 | 141 [132-156] | 35 | 0.075 | 0.204 |
| Diastolic blood pressure, mmHg | 80 [73-89] | 753 | 81 [75-88] | 35 | 0.363 | 0.237 |
| Admission GCS score | 14 [9-15] | 753 | 14 [12-15] | 14 | 0.354 | 0.285 |
| Rotterdam CT score | 2 [2-3] | 749 | 2 [2-2] | 6 | 0.543 | 0.323 |
| **Injury mechanism, n (%)** |  | 720 |  | 21 | 0.103 | 0.504 |
| Traffic accident | 83 (11.5%) |  | 4 (19.0%) |  |  |  |
| Fall from height | 48 (6.7%) |  | 1 (4.8%) |  |  |  |
| Ground-level fall | 93 (12.9%) |  | 6 (28.6%) |  |  |  |
| Other | 496 (68.9%) |  | 10 (47.6%) |  |  |  |

**Notes:** Values are based on observed pre-imputation data. Continuous variables are presented as mean ± SD for age and median [IQR] for other continuous variables; categorical variables are n (%). Percentages use available observations for each variable.

P values were obtained using Welch's t test for age, Mann-Whitney U tests for other continuous variables, and chi-square/Fisher's exact tests for categorical variables, as appropriate. Continuous-variable SMDs were calculated from group means and pooled SDs; categorical SMDs are generalized SMDs.

TRIAGES was calculated only when age, admission GCS, respiratory rate, and systolic blood pressure were all available; a respiratory-rate value of 0 was treated as missing, consistent with the primary analysis.

Pupillary-reactivity and laboratory variables were not formally compared because they were almost entirely unavailable among the 57 excluded patients. The low availability of several baseline prognostic variables in the excluded group should be considered when interpreting between-group comparisons.

**Abbreviations:** CT, computed tomography; GCS, Glasgow Coma Scale; GOS, Glasgow Outcome Scale; IQR, interquartile range; SD, standard deviation; SMD, standardized mean difference; TRIAGES, Trauma Rating Index in Age, Glasgow Coma Scale, Respiratory rate and Systolic blood pressure.

**Supplementary Table S1-2. Extreme-case sensitivity analysis for unavailable discharge GOS**

| **Analysis** | **N** | **Unfavorable functional outcomes, n (%)** | **Adjusted OR per 1-point increase in TRIAGES** | **95% CI** | **P value** |
| --- | --- | --- | --- | --- | --- |
| Primary analysis | 753 | 181 (24.0%) | 2.81 | 2.29-3.45 | <0.001 |
| Best-case scenario: all 57 excluded patients assigned favorable outcome | 810 | 181 (22.3%) | 2.75 | 2.25-3.36 | <0.001 |
| Worst-case scenario: all 57 excluded patients assigned unfavorable outcome | 810 | 238 (29.4%) | 2.33 | 1.97-2.75 | <0.001 |

**Notes:** For the extreme-case scenarios, the 57 patients excluded because discharge GOS was unavailable or invalid were added back to the cohort and were alternatively assigned a favorable functional outcome (best-case scenario) or an unfavorable functional outcome (worst-case scenario).

Missing baseline predictors were handled using the same multiple-imputation-by-chained-equations framework as the primary analysis (20 imputed datasets; predictive mean matching with five nearest donors for continuous variables and multinomial logistic imputation for categorical variables). TRIAGES and the number of nonreactive pupils were re-derived after imputation.

The adjusted model used the same prespecified Model 2 framework as the primary analysis: sex, injury mechanism, number of nonreactive pupils, and Rotterdam CT score; Rotterdam CT score was modeled with the same three-knot restricted cubic spline.

The best- and worst-case analyses are bounding sensitivity analyses and should not be interpreted as assumptions about the true outcomes of the 57 excluded patients.

**Abbreviations:** CI, confidence interval; CT, computed tomography; GCS, Glasgow Coma Scale; GOS, Glasgow Outcome Scale; OR, odds ratio; TRIAGES, Trauma Rating Index in Age, Glasgow Coma Scale, Respiratory rate and Systolic blood pressure.
