# Supplementary material for: Admission TRIAGES score for unfavorable functional outcome after traumatic brain injury: association, prognostic performance, and comparison with the Glasgow coma scale
Source: Front Neurol. 2026 Oct 2;17:1963243. doi: 10.3389/fneur.2026.1963243 (PMC13671951; doi:10.3389/fneur.2026.1963243)
Supplement: Supplementary file 2 [file Supplementary_file_2.DOCX]

**Supplementary Materials S2: TRIAGES Scoring Algorithm and Implementation**

**Supplementary Methods**

**TRIAGES scoring algorithm**

The TRIAGES score was calculated according to the original algorithm developed by Shiraishi et al. (1). The score is the unweighted sum of four component scores for age, Glasgow Coma Scale (GCS), respiratory rate, and systolic blood pressure (SBP). Age contributes 0-2 points, GCS 0-6 points, respiratory rate 0-2 points, and SBP 0-4 points, yielding a theoretical total score of 0-14. Higher scores indicate greater physiological and neurological derangement. No component weights or category boundaries were re-estimated in the present study.

**Supplementary Table S2-1. Original TRIAGES point-assignment algorithm**

| **Component** | **Observed value/range** | **Points** |
| --- | --- | --- |
| **Age, years** | 16–54 | 0 |
|  | 55–74 | 1 |
|  | ≥75 | 2 |
| **GCS** | 15 | 0 |
|  | 14 | 1 |
|  | 12–13 | 2 |
|  | 8–11 | 3 |
|  | 5–7 | 4 |
|  | 4 | 5 |
|  | 3 | 6 |
| **Respiratory rate, breaths/min** | 12–27 | 0 |
|  | 4–11 | 1 |
|  | ≥28 | 1 |
|  | 0–3 | 2 |
| **SBP, mmHg** | 100–199 | 0 |
|  | 80–99 | 1 |
|  | ≥200 | 1 |
|  | 50–79 | 2 |
|  | 0–49 | 4 |

**Total TRIAGES score = age points + GCS points + respiratory-rate points + SBP points; theoretical range, 0–14. The age categories shown in this table reproduce the original TRIAGES scoring algorithm. In the original derivation study, patients aged <16 years were excluded and the reference age category was 16–54 years. The present study applied a separate eligibility criterion of age >18 years; therefore, patients aged 16–18 years were not included. This study-specific eligibility criterion did not alter the original TRIAGES scoring categories or point assignments.**

**Boundary values and implementation**

All boundary values were assigned inclusively to the categories shown in Supplementary Table S2-1, without interpolation. The point assignments and category boundaries were implemented as published in the original TRIAGES derivation study. In the present cohort, TRIAGES was derived from age and the earliest available GCS, respiratory rate, and SBP documented after arrival at the study emergency department and before therapeutic intervention initiated at the study hospital.

**Handling of missing and implausible values**

Prespecified data-quality checks were performed before statistical modeling. Structurally invalid or clearly implausible component values were treated as missing rather than assigned a TRIAGES category. No partial TRIAGES score was calculated from an incomplete set of components. In the multiple-imputation analyses, missing component variables were imputed first and TRIAGES was then re-derived in each completed dataset, thereby preserving the published scoring rule.

**Handling of a respiratory-rate value of 0**

One recorded respiratory-rate value of 0 was treated as missing in the primary analysis because it was considered a data-quality concern. In a prespecified sensitivity analysis, this value was retained and scored according to the original TRIAGES algorithm, under which a respiratory rate of 0–3 breaths/min receives 2 points. The adjusted association was unchanged (OR 2.81, 95% CI 2.29–3.45), supporting the robustness of the primary result to this data-processing decision.

**Supplementary Results**

In the 753-patient analytic dataset used for descriptive presentation, TRIAGES scores ranged from 0 to 10, although the theoretical scale ranges from 0 to 14. Scores of 11–14 were not observed. All age-point categories (0–2), all GCS-point categories (0–6), and all respiratory-rate point categories (0–2) were represented. For SBP, point categories 0, 1, and 2 were represented, whereas the 4-point category corresponding to SBP 0–49 mmHg was not observed.

**Supplementary Table S2-2. Distribution of total TRIAGES scores in the 753-patient analytic dataset**

| **TRIAGES score** | **n** | **%** |
| --- | --- | --- |
| 0 | 120 | 15.9 |
| 1 | 186 | 24.7 |
| 2 | 103 | 13.7 |
| 3 | 105 | 13.9 |
| 4 | 110 | 14.6 |
| 5 | 67 | 8.9 |
| 6 | 46 | 6.1 |
| 7 | 11 | 1.5 |
| 8 | 3 | 0.4 |
| 9 | 1 | 0.1 |
| 10 | 1 | 0.1 |
| 11 | 0 | 0.0 |
| 12 | 0 | 0.0 |
| 13 | 0 | 0.0 |
| 14 | 0 | 0.0 |

**Supplementary Table S2-3. Distribution of TRIAGES component points in the 753-patient analytic dataset**

| **Component** | **Points** | **n** | **%** |
| --- | --- | --- | --- |
| **Age points** | 0 | 373 | 49.5 |
|  | 1 | 300 | 39.8 |
|  | 2 | 80 | 10.6 |
| **GCS points** | 0 | 267 | 35.5 |
|  | 1 | 118 | 15.7 |
|  | 2 | 106 | 14.1 |
|  | 3 | 101 | 13.4 |
|  | 4 | 105 | 13.9 |
|  | 5 | 29 | 3.9 |
|  | 6 | 27 | 3.6 |
| **Respiratory-rate points** | 0 | 746 | 99.1 |
|  | 1 | 6 | 0.8 |
|  | 2 | 1 | 0.1 |
| **SBP points** | 0 | 704 | 93.5 |
|  | 1 | 48 | 6.4 |
|  | 2 | 1 | 0.1 |
|  | 4 | 0 | 0.0 |

**Note:** The distribution tables summarize the 753-patient analytic dataset supplied for the present study. Percentages may not total exactly 100.0 because of rounding.

**Abbreviations**

GCS, Glasgow Coma Scale; OR, odds ratio; SBP, systolic blood pressure; TRIAGES, Trauma Rating Index in Age, Glasgow Coma Scale, Respiratory rate and Systolic blood pressure.

**References**

1.	Shiraishi A, Otomo Y, Yoshikawa S, Morishita K, Roberts I, Matsui H. Derivation and validation of an easy-to-compute trauma score that improves prognostication of mortality or the trauma rating index in age, glasgow coma scale, respiratory rate and systolic blood pressure (TRIAGES) score. *Crit Care* (2019) 23:365. doi: 10.1186/s13054-019-2636-x
