# Supplementary material for: Admission TRIAGES score for unfavorable functional outcome after traumatic brain injury: association, prognostic performance, and comparison with the Glasgow coma scale
Source: Front Neurol. 2026 Oct 2;17:1963243. doi: 10.3389/fneur.2026.1963243 (PMC13671951; doi:10.3389/fneur.2026.1963243)
Supplement: Supplementary file 3 [file Supplementary_file_3.DOCX]

**Data Quality Assessment, Missingness Profile, Coding Validation, and TRIAGES Score Verification**

**S1.1 Scope and data-quality procedures**

The source spreadsheet contained 998 traumatic brain injury records and 116 fields. Data quality assessment was performed before model fitting and included duplicate-record reconciliation, eligibility verification, missingness profiling, structural coding checks, range and internal-consistency checks, and independent recalculation of the TRIAGES score. Blank cells and whitespace-only entries were treated as missing. Patient names were not carried into the analytic dataset; analysis_id and source_excel_row were retained solely for traceability and were excluded from statistical models.

Duplicate hospitalization identifiers were reconciled before cohort selection. Each duplicated identifier occurred twice. The later record was retained under a deterministic reconciliation rule, which was consistent with the existing analytic file. Missingness was quantified in the cleaned, pre-imputation cohort. The fully populated working CSV was not used to estimate missingness. Covariate missingness is intended to be addressed using multiple imputation in the formal analysis; the outcome will not be imputed, and TRIAGES will be recalculated within each imputed dataset.

Plausibility rules were used as data-audit thresholds rather than clinical exclusion criteria. Values that violated structural codes or clear prespecified plausibility limits were converted to missing rather than winsorized. Extreme values that remained within the audit limits were retained. This audit assessed the electronic source fields and internal consistency; it did not independently adjudicate whether admission measurements preceded prehospital intubation, sedation, or resuscitation.

**Supplementary Table S1A. Cohort construction and database reconciliation**

| **Processing step** | **n** | **Definition/decision** |
| --- | --- | --- |
| Raw TBI records | 998 | Source spreadsheet |
| Duplicated hospitalization-ID groups | 45 groups (90 records) | Each duplicated identifier occurred twice |
| Unique patients after duplicate reconciliation | 953 | One record retained per hospitalization identifier |
| Age criterion not met | 143 | 142 patients aged <=18 years and 1 patient with missing age |
| Discharge GOS missing or invalid | 69 | 67 missing values and 2 records coded as GOS = 0 |
| Patients meeting both exclusion criteria | 12 | Overlap; not double-counted |
| Total excluded after reconciliation | 200 | 131 age criterion only, 57 GOS criterion only, and 12 both |
| Final adult TBI cohort | 753 | Age >18 years and valid discharge GOS 1-5 |
| Unfavorable discharge GOS | 181 (24.0%) | GOS 1-3 |
| Favorable discharge GOS | 572 (76.0%) | GOS 4-5 |

**Note: Exclusion counts for age and GOS overlap; therefore, the individual criterion counts do not sum directly to 200.**

**Supplementary Table S1B. Variable-level missingness before imputation**

| **Variable** | **Unit/coding** | **Source missing, n** | **Additional invalid values set missing, n** | **Final pre-imputation missing, n (%)** | **Favorable GOS missing, n (%)** | **Unfavorable GOS missing, n (%)** |
| --- | --- | --- | --- | --- | --- | --- |
| Unfavorable discharge GOS | binary (GOS 1-3 vs 4-5) | 0 | 0 | 0 (0.0) | 0 (0.0) | 0 (0.0) |
| Age | years | 0 | 0 | 0 (0.0) | 0 (0.0) | 0 (0.0) |
| Sex | male/female | 0 | 0 | 0 (0.0) | 0 (0.0) | 0 (0.0) |
| Body temperature | degrees C | 0 | 0 | 0 (0.0) | 0 (0.0) | 0 (0.0) |
| Respiratory rate | breaths/min | 0 | 1 | 1 (0.1) | 0 (0.0) | 1 (0.6) |
| Heart rate | beats/min | 0 | 1 | 1 (0.1) | 0 (0.0) | 1 (0.6) |
| Systolic blood pressure | mmHg | 0 | 0 | 0 (0.0) | 0 (0.0) | 0 (0.0) |
| Diastolic blood pressure | mmHg | 0 | 2 | 2 (0.3) | 0 (0.0) | 2 (1.1) |
| Admission GCS | points | 0 | 0 | 0 (0.0) | 0 (0.0) | 0 (0.0) |
| Left pupillary reactivity | 0/1/2 | 62 | 0 | 62 (8.2) | 43 (7.5) | 19 (10.5) |
| Right pupillary reactivity | 0/1/2 | 43 | 0 | 43 (5.7) | 28 (4.9) | 15 (8.3) |
| Rotterdam CT score | points | 4 | 0 | 4 (0.5) | 2 (0.3) | 2 (1.1) |
| Injury mechanism | categories 1-4 | 33 | 0 | 33 (4.4) | 20 (3.5) | 13 (7.2) |
| White blood cell count | 10^9/L | 27 | 2 | 29 (3.9) | 22 (3.8) | 7 (3.9) |
| Absolute neutrophil count | 10^9/L | 27 | 2 | 29 (3.9) | 22 (3.8) | 7 (3.9) |
| Absolute lymphocyte count | 10^9/L | 27 | 2 | 29 (3.9) | 22 (3.8) | 7 (3.9) |
| Absolute monocyte count | 10^9/L | 27 | 2 | 29 (3.9) | 22 (3.8) | 7 (3.9) |
| RDW-CV | % | 27 | 2 | 29 (3.9) | 22 (3.8) | 7 (3.9) |
| Platelet count | 10^9/L | 27 | 2 | 29 (3.9) | 22 (3.8) | 7 (3.9) |
| Blood glucose | mmol/L | 30 | 0 | 30 (4.0) | 24 (4.2) | 6 (3.3) |
| Serum potassium | mmol/L | 30 | 0 | 30 (4.0) | 24 (4.2) | 6 (3.3) |
| Fibrinogen | g/L | 76 | 1 | 77 (10.2) | 64 (11.2) | 13 (7.2) |

**Note: Percentages in the outcome-specific columns use n = 572 for favorable GOS and n = 181 for unfavorable GOS. Source missingness was evaluated after duplicate reconciliation and eligibility screening. Additional invalid values were identified during quality control and converted to missing. GCS, Glasgow Coma Scale; GOS, Glasgow Outcome Scale; RDW-CV, red-cell distribution width coefficient of variation.**

**Supplementary Table S1C. Range, coding, and internal-consistency checks**

| **Field/domain** | **Validation rule** | **Observed finding** | **Affected records** | **Database-freeze decision** |
| --- | --- | --- | --- | --- |
| Hospitalization identifier | One independent record per identifier | 45 identifiers occurred twice; no identifier occurred more than twice | 45 groups | Later record retained; 953 unique patients remained |
| Duplicate-record conflicts | Agreement expected across duplicate entries | Discordance occurred for discharge GOS in 3 groups and Rotterdam CT score in 7 groups; no discordance in other audited study variables | 3 and 7 groups | Resolved using the later record under the deterministic reconciliation rule |
| Age and discharge GOS | Age >18 years; GOS coded 1-5 | 142 patients aged <=18 years; 1 age missing; 67 GOS missing; 2 GOS coded 0 | 200 unique patients excluded | Applied eligibility criteria before analysis |
| Sex | Original code 1 = male, 2 = female | All 753 included records had a valid code | 0 invalid | Recoded to sex_male: 1 = male, 0 = female |
| Body temperature | Audit range 30-45 degrees C | 36.0-39.4 degrees C | 0 outside range | Retained |
| Respiratory rate | Positive value; audit range 1-80 breaths/min | One value of 0; remaining range 3-57 | 1 | Zero treated as a placeholder and converted to missing before imputation |
| Heart rate | Audit range 20-250 beats/min | One value of 19; cleaned range 38-164 | 1 | Value converted to missing before imputation |
| Systolic blood pressure | Audit range 30-300 mmHg | 58-261 mmHg | 0 outside range | Retained; no automatic truncation or winsorization |
| Diastolic blood pressure | Audit range 20-200 mmHg | Values of 12 and 16; cleaned range 34-167 | 2 | Values converted to missing before imputation |
| Admission GCS | Integer code 3-15 | 3-15 | 0 invalid | Retained |
| Pupillary reactivity | Each side coded 0, 1, or 2 | All observed values valid; 62 left-side and 43 right-side values missing | 0 invalid codes | Missing values retained for multiple imputation |
| Rotterdam CT score | Integer code 1-6 | All observed values valid; 4 missing | 0 invalid codes | Missing values retained for multiple imputation |
| Injury mechanism | Code 1-4 | All observed values valid; 33 missing | 0 invalid codes | Treated as a nominal categorical variable; missing values retained for imputation |
| CBC-derived study variables | Cross-field internal consistency and prespecified broad ranges | Two records showed mutually inconsistent WBC/differential/RDW/platelet patterns | 2 records | WBC, absolute neutrophil, lymphocyte, monocyte, RDW-CV, and platelet values set to missing in both records |
| Blood glucose | Positive numeric value; broad audit range 0.5-60 mmol/L | 3.65-34.01 mmol/L | 0 outside range | Retained |
| Serum potassium | Broad audit range 1.5-8.0 mmol/L | 2.21-6.00 mmol/L | 0 outside range | Retained |
| Fibrinogen | Positive value; audit range 0.1-10.0 g/L | One value of 0 and one apparent decimal-place value of 24.2; cleaned range 0.51-6.70 | 2 values reviewed | 0 converted to missing; 24.2 standardized to 2.42 g/L based on the recorded unit and the existing cleaned dataset |

**Note: Audit ranges were prespecified for error detection and were not used as clinical eligibility criteria. Values within the stated audit limits were retained even when clinically extreme. The patient-level correction log is maintained internally and is not included in the public supplementary file.**

**Supplementary Table S1D. Independent recalculation and consistency of the TRIAGES score**

| **Score element** | **Recalculation rule** | **Observed range** | **Agreement with source value** | **Agreement with stored component/score** |
| --- | --- | --- | --- | --- |
| Age component | 16-54 = 0; 55-74 = 1; >=75 = 2 | 0-2 | 753/753 (100.0%) | 753/753 (100.0%) |
| GCS component | 15 = 0; 14 = 1; 12-13 = 2; 8-11 = 3; 5-7 = 4; 4 = 5; 3 = 6 | 0-6 | 753/753 (100.0%) | 753/753 (100.0%) |
| Respiratory-rate component | 0-3 = 2; 4-11 = 1; 12-27 = 0; >=28 = 1 | 0-2 | 752/753 (99.9%) | 753/753 (100.0%) |
| Systolic-BP component | 0-49 = 4; 50-79 = 2; 80-99 = 1; 100-199 = 0; >=200 = 1 | 0-2 | 753/753 (100.0%) | 753/753 (100.0%) |
| TRIAGES total score | Sum of the four components; theoretical range 0-14 | 0-10 | Not applicable | 753/753 (100.0%) |

**Note: The single respiratory-rate disagreement reflects the value of 0 identified during quality control; the current fully populated working dataset contains 20 breaths/min for that record. After applying the stored cleaned values, every TRIAGES component and total score was reproduced exactly. The observed TRIAGES median was 2 (interquartile range, 1-4), and the observed total-score range was 0-10. BP, blood pressure; GCS, Glasgow Coma Scale.**

**S1.2 Database-freeze statement**

The database-freeze cohort contains 753 unique adult patients, including 181 unfavorable discharge outcomes and 572 favorable discharge outcomes. The primary outcome, age, sex, admission GCS, and systolic blood pressure were complete. The maximum pre-imputation missingness was 10.2% for fibrinogen. All data-quality decisions described above were applied before statistical modeling. The frozen pre-imputation dataset should be used for multiple imputation and sensitivity analyses, while the existing fully populated CSV may be retained only as a preliminary working dataset.

**S1.3 Abbreviations**

BP, blood pressure; CBC, complete blood count; CT, computed tomography; GCS, Glasgow Coma Scale; GOS, Glasgow Outcome Scale; RDW-CV, red-cell distribution width coefficient of variation; TBI, traumatic brain injury; TRIAGES, TRIAGES score.
