# Supplementary material for: Admission TRIAGES score for unfavorable functional outcome after traumatic brain injury: association, prognostic performance, and comparison with the Glasgow coma scale
Source: Front Neurol. 2026 Oct 2;17:1963243. doi: 10.3389/fneur.2026.1963243 (PMC13671951; doi:10.3389/fneur.2026.1963243)
Supplement: Supplementary file 4 [file Supplementary_file_4.DOCX]

**Sensitivity and Subgroup Analyses of the Association Between the TRIAGES Score and Discharge Outcomes**

**S4.1 Scope and analytical approach**

Sensitivity analyses assessed the robustness of the association between the TRIAGES score and unfavorable functional status at hospital discharge. The prespecified primary model estimated the adjusted odds ratio (OR) per 1-point increase in TRIAGES and used the same final Model 2 specification as Table 2, Table 3, and Supplementary Material S3: sex, injury mechanism, the number of nonreactive pupils, and Rotterdam CT score, with Rotterdam CT score modeled using a three-knot restricted cubic spline with knots at scores 1, 2, and 5. Missing covariate data in the primary analysis were handled using multiple imputation by chained equations with 20 completed datasets, and estimates were combined using Rubin's rules. Complete-case and single-imputation analyses were retained as sensitivity analyses.

Additional analyses used mRS 3-6 as an alternative binary outcome, modeled the original GOS categories using ordinal logistic regression, excluded patients who died in hospital, retained the single source respiratory-rate value of 0 as observed, excluded patients recorded as intubated at admission, and restricted the cohort to isolated TBI. The available database did not distinguish prehospital intubation from sedation or intubation performed after arrival; therefore, the relevant sensitivity analysis is described only as exclusion of patients intubated at admission. Subgroup analyses were performed by sex, age (<65 vs ≥65 years), and admission GCS severity. For the primary imputed analyses, subgroup-specific estimates and interaction tests were obtained using the same final Model 2 specification within each imputed dataset and combined across imputations.

**Supplementary Table S4A. Sensitivity analyses of the association between the TRIAGES score and discharge outcomes**

| **Analysis** | **Outcome definition / analysis population** | **n** | **Events** | **Adjusted OR per 1-point increase** | **95% CI** | **P value** |
| --- | --- | --- | --- | --- | --- | --- |
| Primary analysis (MICE) | Unfavorable discharge GOS (1-3); 20 multiply imputed datasets | 753 | 181 | 2.81 | 2.29-3.45 | <0.001 |
| Complete-case analysis | Unfavorable discharge GOS (1-3); patients complete for all model variables | 689 | 160 | 2.76 | 2.24-3.40 | <0.001 |
| Single-imputation analysis | Unfavorable discharge GOS (1-3); fully populated working dataset | 753 | 181 | 2.86 | 2.33-3.50 | <0.001 |
| Alternative binary outcome: mRS 3-6 | Unfavorable discharge mRS (3-6) | 753 | 183 | 2.54 | 2.10-3.07 | <0.001 |
| Ordinal logistic model for GOS 1-5 | Original GOS 1-5; common OR for a worse GOS category | 753 | — | 1.97 | 1.75-2.22 | <0.001 |
| Excluding death: GOS 2-3 vs 4-5 | Patients with GOS 2-5; GOS 2-3 vs GOS 4-5 | 692 | 120 | 3.06 | 2.41-3.90 | <0.001 |
| Respiratory rate 0 treated as observed | Single source value of 0 retained and TRIAGES recalculated | 753 | 181 | 2.81 | 2.29-3.45 | <0.001 |
| Excluding intubated at admission | Patients not recorded as intubated at admission | 742 | 171 | 2.78 | 2.26-3.41 | <0.001 |
| Isolated TBI only | Patients without associated injury to another organ system | 627 | 138 | 2.99 | 2.33-3.84 | <0.001 |

**Note: Except for the explicitly labeled complete-case and single-imputation analyses, estimates were obtained under the final Model 2 framework and, where applicable, pooled across the 20 imputed datasets using Rubin's rules. Model 2 adjusted for sex, injury mechanism, the number of nonreactive pupils, and Rotterdam CT score, with Rotterdam CT score modeled using a three-knot restricted cubic spline with knots at 1, 2, and 5. The ordinal logistic model reports a common OR for a worse GOS category. Events are not shown for the ordinal outcome because all five GOS categories contributed to the model. mRS, modified Rankin Scale.**

**Supplementary Table S4B. Subgroup analyses of the association between the TRIAGES score and unfavorable discharge GOS**

| **Subgroup** | **Category** | **n** | **Events** | **Adjusted OR per 1-point increase** | **95% CI** | **P value** | **P for interaction** |
| --- | --- | --- | --- | --- | --- | --- | --- |
| Sex | Female | 241 | 50 | 4.51 | 2.53-8.05 | <0.001 | 0.039 |
|  | Male | 512 | 131 | 2.55 | 2.04-3.19 | <0.001 |  |
| Age | <65 years | 550 | 109 | 2.71 | 2.10-3.49 | <0.001 | 0.334 |
|  | ≥65 years | 203 | 72 | 2.92 | 1.95-4.38 | <0.001 |  |
| Admission GCS | Mild (13-15) | 424 | 18 | 3.33 | 2.03-5.46 | <0.001 | 0.931 |
|  | Moderate (9-12) | 141 | 42 | 4.15 | 2.10-8.20 | <0.001 |  |
|  | Severe (3-8) | 188 | 121 | 2.87 | 1.88-4.39 | <0.001 |  |

**Note: Subgroup-specific estimates represent adjusted ORs per 1-point increase in the TRIAGES score and were pooled across the 20 imputed datasets using the final Model 2 specification. The sex-stratified models omitted sex from the adjustment set; other models retained the prespecified covariates when estimable. Interaction P values were obtained from the corresponding TRIAGES-by-subgroup interaction models and combined across imputations. The sex interaction was statistically significant (P for interaction = 0.039), whereas interactions with age and admission GCS severity were not significant. Subgroup analyses were exploratory, and no multiplicity adjustment was applied.**

**S4.2 Summary interpretation**

The association between higher TRIAGES scores and worse discharge outcomes remained statistically significant across all feasible sensitivity analyses after harmonizing the covariate specification to the final Model 2. Effect estimates remained similar across the primary MICE, complete-case, and single-imputation analyses and were robust to alternative outcome definitions and restricted populations. A potential interaction by sex was observed (P for interaction = 0.039); because this was an exploratory subgroup analysis without multiplicity adjustment, it should be interpreted cautiously and requires external confirmation.

**S4.3 Abbreviations**

CI, confidence interval; CT, computed tomography; GCS, Glasgow Coma Scale; GOS, Glasgow Outcome Scale; MICE, multiple imputation by chained equations; mRS, modified Rankin Scale; OR, odds ratio; TBI, traumatic brain injury; TRIAGES, Trauma Rating Index in Age, Glasgow Coma Scale, Respiratory rate and Systolic blood pressure.
