# Supplementary material for: Admission TRIAGES score for unfavorable functional outcome after traumatic brain injury: association, prognostic performance, and comparison with the Glasgow coma scale
Source: Front Neurol. 2026 Oct 2;17:1963243. doi: 10.3389/fneur.2026.1963243 (PMC13671951; doi:10.3389/fneur.2026.1963243)
Supplement: Supplementary file 5 [file Supplementary_file_5.DOCX]

**Multiple Imputation, Diagnostic Assessment, and Sensitivity Analyses**

**S2.1 Rationale and primary missing-data strategy**

Missing covariate data were handled using multiple imputation by chained equations under a missing-at-random assumption conditional on the variables included in the imputation model. The primary outcome, unfavorable functional status at hospital discharge, was complete and was not imputed. Patient identifiers and source-row identifiers were excluded from the imputation model. All non-derived study variables, including the outcome, were available as predictors in the conditional models.

Continuous variables were imputed using predictive mean matching with five nearest donors. Categorical variables with missing values were imputed using multinomial logistic regression. Parameter uncertainty was introduced by bootstrap resampling of observed cases at each conditional update. After 10 burn-in cycles, 20 completed datasets were retained at two-cycle intervals from a total of 50 chained-equation cycles. The random seed was 20260806.

The four original TRIAGES inputs-age, admission Glasgow Coma Scale score, respiratory rate, and systolic blood pressure-were treated as the source variables. TRIAGES components and the total score were recalculated passively within each completed dataset; the TRIAGES total score was not directly imputed. The number of nonreactive pupils was similarly re-derived from the imputed left- and right-pupil variables. Regression estimates and covariance matrices were combined using Rubin rules.

| **Key item** | **Result** |
| --- | --- |
| Maximum variable-level missingness | 10.2% (fibrinogen) |
| Number of imputations | 20 |
| Adjusted complete-case sample | 689/753 patients (91.5%) |
| Primary MICE result | Adjusted OR per 1-point TRIAGES increase: 2.81 (2.29-3.45) |
| Sensitivity consistency | Complete-case OR 2.76; existing single-imputed OR 2.86 |

**S2.2 Imputation specification**

**Supplementary Table S2A. Prespecified multiple-imputation framework**

| **Component** | **Specification** | **Implementation detail** |
| --- | --- | --- |
| Analysis population | 753 adult patients with TBI; 181 unfavorable discharge GOS events | The outcome was complete and was not imputed. |
| Imputation framework | Multiple imputation by chained equations using fully conditional specification | Twenty completed datasets were retained. |
| Continuous variables | Predictive mean matching with five nearest donors | Observed donor values were used, preserving plausible ranges and distributional shape. |
| Categorical variables | Multinomial logistic regression | Applied to pupillary reactivity, Rotterdam CT score, and injury mechanism. |
| Parameter uncertainty | Bootstrap resampling of observed cases at each conditional update | Used within each chained-equation cycle. |
| Predictor matrix | All other non-derived study variables, including the outcome | Patient identifiers and source-row identifiers were excluded. |
| Iteration schedule | 10 burn-in cycles; 20 datasets retained at two-cycle intervals; 50 cycles in total | Random seed: 20260806. |
| Passive derivation | TRIAGES components and total score recalculated after imputation of the original variables | TRIAGES total score was never directly imputed. |
| Pooling | Regression coefficients and covariance matrices combined using Rubin rules | Total variance included within- and between-imputation components. |
| Sensitivity analyses | Complete-case analysis and the existing fully populated preliminary dataset | The multiple-imputation analysis was prespecified as the primary approach. |

**Note: MICE denotes multiple imputation by chained equations. The outcome was included as a predictor to preserve associations but was never imputed.**

**Supplementary Table S2B. Variables imputed and conditional methods**

| **Variable** | **Unit/coding** | **Missing, n (%)** | **Conditional imputation method** | **Passive derivation/analytic role** |
| --- | --- | --- | --- | --- |
| Fibrinogen | g/L | 77 (10.2) | Predictive mean matching (k = 5) | Covariate/predictor |
| Left pupillary reactivity | 0/1/2 | 62 (8.2) | Multinomial logistic regression | Used to derive number of nonreactive pupils after imputation |
| Right pupillary reactivity | 0/1/2 | 43 (5.7) | Multinomial logistic regression | Used to derive number of nonreactive pupils after imputation |
| Injury mechanism | 1-4 | 33 (4.4) | Multinomial logistic regression | Covariate/predictor |
| Blood glucose | mmol/L | 30 (4.0) | Predictive mean matching (k = 5) | Covariate/predictor |
| Serum potassium | mmol/L | 30 (4.0) | Predictive mean matching (k = 5) | Covariate/predictor |
| White blood cell count | 10^9/L | 29 (3.9) | Predictive mean matching (k = 5) | Covariate/predictor |
| Absolute neutrophil count | 10^9/L | 29 (3.9) | Predictive mean matching (k = 5) | Covariate/predictor |
| Absolute lymphocyte count | 10^9/L | 29 (3.9) | Predictive mean matching (k = 5) | Covariate/predictor |
| Absolute monocyte count | 10^9/L | 29 (3.9) | Predictive mean matching (k = 5) | Covariate/predictor |
| RDW-CV | % | 29 (3.9) | Predictive mean matching (k = 5) | Covariate/predictor |
| Platelet count | 10^9/L | 29 (3.9) | Predictive mean matching (k = 5) | Covariate/predictor |
| Rotterdam CT score | 1-6 | 4 (0.5) | Multinomial logistic regression | Covariate/predictor |
| Diastolic blood pressure | mmHg | 2 (0.3) | Predictive mean matching (k = 5) | Covariate/predictor |
| Respiratory rate | breaths/min | 1 (0.1) | Predictive mean matching (k = 5) | Original TRIAGES component; score recalculated passively |
| Heart rate | beats/min | 1 (0.1) | Predictive mean matching (k = 5) | Covariate/predictor |

**Note: Age, sex, admission GCS, systolic blood pressure, body temperature, and the primary outcome were complete and therefore required no imputation. Sex and the outcome were nevertheless included as predictors in the conditional models.**

**S2.3 Distributional and convergence diagnostics**

Imputed values remained within the observed donor ranges for every continuous variable because predictive mean matching was used. For variables with more than 3% missingness, the observed and imputed distributions were similar. Late-cycle summaries showed stable fluctuation without sustained directional drift. Variables with only one to four missing observations exhibited greater stochastic variation in their cycle-specific means, as expected, but had negligible influence on the overall cohort distributions.

**Supplementary Table S2C. Observed and pooled imputed distributions for continuous variables**

| **Variable** | **Unit** | **Missing, n (%)** | **Observed median [IQR]** | **Pooled imputed median [IQR]** | **Observed range** | **Imputed range** |
| --- | --- | --- | --- | --- | --- | --- |
| Fibrinogen | g/L | 77 (10.2) | 2.32 [1.85-2.98] | 2.29 [1.87-2.94] | 0.51-6.70 | 0.51-6.70 |
| Blood glucose | mmol/L | 30 (4.0) | 7.59 [6.44-9.28] | 7.20 [6.13-8.82] | 3.65-34.01 | 3.65-34.01 |
| Serum potassium | mmol/L | 30 (4.0) | 3.56 [3.26-3.91] | 3.60 [3.30-3.92] | 2.21-6.00 | 2.38-6.00 |
| White blood cell count | 10^9/L | 29 (3.9) | 13.45 [10.08-17.41] | 14.01 [10.38-19.90] | 1.80-49.95 | 6.80-23.90 |
| Absolute neutrophil count | 10^9/L | 29 (3.9) | 10.64 [7.20-14.90] | 12.09 [8.50-16.00] | 1.50-41.55 | 4.60-22.17 |
| Absolute lymphocyte count | 10^9/L | 29 (3.9) | 1.50 [0.98-2.40] | 1.20 [0.90-2.07] | 0.20-9.74 | 0.30-6.57 |
| Absolute monocyte count | 10^9/L | 29 (3.9) | 0.71 [0.50-1.01] | 0.80 [0.50-1.20] | 0.06-4.33 | 0.10-2.48 |
| RDW-CV | % | 29 (3.9) | 12.90 [12.40-13.50] | 12.80 [12.50-13.40] | 11.20-23.90 | 11.20-22.40 |
| Platelet count | 10^9/L | 29 (3.9) | 222.00 [183.00-262.25] | 232.00 [198.00-272.25] | 24.00-672.00 | 39.00-672.00 |
| Diastolic blood pressure | mmHg | 2 (0.3) | 80.00 [73.00-89.00] | 107.00 [98.00-129.00] | 34.00-167.00 | 77.00-167.00 |
| Respiratory rate | breaths/min | 1 (0.1) | 20.00 [20.00-21.00] | 20.50 [20.00-21.25] | 3.00-57.00 | 16.00-40.00 |
| Heart rate | beats/min | 1 (0.1) | 81.00 [74.00-90.00] | 93.00 [81.00-104.25] | 38.00-164.00 | 66.00-130.00 |

**Note: Pooled imputed summaries combine the imputed cells from all 20 completed datasets. Observed summaries use nonmissing values from the frozen pre-imputation dataset. IQR, interquartile range.**

**Supplementary Table S2D. Observed and pooled imputed category distributions**

| **Variable** | **Missing, n (%)** | **Observed category distribution** | **Pooled imputed category distribution** |
| --- | --- | --- | --- |
| Left pupillary reactivity | 62 (8.2) | 0: 80.8%; 1: 8.1%; 2: 11.1% | 0: 80.6%; 1: 7.3%; 2: 12.1% |
| Right pupillary reactivity | 43 (5.7) | 0: 80.7%; 1: 7.2%; 2: 12.1% | 0: 73.0%; 1: 8.1%; 2: 18.8% |
| Injury mechanism | 33 (4.4) | 1: 11.5%; 2: 6.7%; 3: 12.9%; 4: 68.9% | 1: 11.8%; 2: 9.7%; 3: 10.9%; 4: 67.6% |
| Rotterdam CT score | 4 (0.5) | 1: 23.1%; 2: 35.0%; 3: 21.4%; 4: 9.7%; 5: 6.0%; 6: 4.8% | 1: 5.0%; 2: 20.0%; 3: 27.5%; 4: 15.0%; 5: 21.2%; 6: 11.2% |

**Note: Coding: pupillary reactivity, 0 = brisk, 1 = sluggish, and 2 = absent/nonreactive; injury mechanism, 1 = traffic accident, 2 = fall from height, 3 = ground-level fall, and 4 = other; Rotterdam CT score, 1-6.**

**Supplementary Table S2E. Late-cycle convergence assessment for variables with at least 29 missing observations**

| **Variable** | **Missing, n** | **Standardized late-cycle drift** | **Assessment** |
| --- | --- | --- | --- |
| Fibrinogen | 77 | 0.062 | No material late-cycle drift |
| Left pupillary reactivity | 62 | 0.029 | No material late-cycle drift |
| Right pupillary reactivity | 43 | 0.007 | No material late-cycle drift |
| Injury mechanism | 33 | 0.029 | No material late-cycle drift |
| Blood glucose | 30 | 0.100 | Small late-cycle fluctuation; no monotonic drift observed |
| Serum potassium | 30 | 0.049 | No material late-cycle drift |
| White blood cell count | 29 | 0.006 | No material late-cycle drift |
| Absolute neutrophil count | 29 | 0.006 | No material late-cycle drift |
| Absolute lymphocyte count | 29 | 0.037 | No material late-cycle drift |
| Absolute monocyte count | 29 | 0.091 | No material late-cycle drift |
| RDW-CV | 29 | 0.075 | No material late-cycle drift |
| Platelet count | 29 | 0.060 | No material late-cycle drift |

**Note: Standardized late-cycle drift was calculated as the absolute difference between the mean imputed value during the final five cycles and the preceding five cycles, divided by the observed standard deviation. Values near zero indicate no material directional drift. For categorical variables, the numeric coding was used only for this convergence summary.**

**S2.4 Rubin-pooled analysis and sensitivity analyses**

The primary association analyses were fitted separately in each of the 20 completed datasets and combined using Rubin rules. The crude model included TRIAGES as the sole predictor. The adjusted model used the same prespecified Model 2 specification as the main analysis: TRIAGES, sex, injury mechanism, number of nonreactive pupils, and Rotterdam CT score, with Rotterdam CT score modeled using a three-knot restricted cubic spline with knots at scores 1, 2, and 5. Results were compared with complete-case analyses and with the existing fully populated preliminary working dataset.

**Supplementary Table S2F Sensitivity of the TRIAGES association to the missing-data approach**

| **Missing-data approach** | **Model** | **Patients / events** | **OR per 1-point increase (95% CI)** | **P value** | **AUC** |
| --- | --- | --- | --- | --- | --- |
| MICE, primary analysis | Crude | 753 / 181 | 3.13 (2.63-3.72) | <0.001 | 0.905 |
| Complete cases | Crude | 752 / 180 | 3.12 (2.63-3.71) | <0.001 | 0.905 |
| Existing single-imputed dataset | Crude | 753 / 181 | 3.13 (2.63-3.72) | <0.001 | 0.905 |
| MICE, primary analysis | Adjusted | 753 / 181 | 2.81 (2.29-3.45) | <0.001 | 0.921 |
| Complete cases | Adjusted | 689 / 160 | 2.76 (2.24-3.40) | <0.001 | 0.916 |
| Existing single-imputed dataset | Adjusted | 753 / 181 | 2.86 (2.33-3.50) | <0.001 | 0.920 |

**Note: The adjusted model used the final prespecified Model 2 specification: TRIAGES, sex, injury mechanism, number of nonreactive pupils, and Rotterdam CT score; Rotterdam CT score was modeled using a three-knot restricted cubic spline with knots at 1, 2, and 5. For the multiple-imputation analyses, AUC is the arithmetic mean across the 20 completed datasets and is reported descriptively; regression coefficients and confidence intervals were pooled using Rubin rules. CI, confidence interval; OR, odds ratio.**

**S2.5 Summary**

Multiple imputation retained all 753 patients and yielded an adjusted odds ratio of 2.81 (95% CI, 2.29-3.45) for each 1-point increase in TRIAGES under the final Model 2 specification, in which Rotterdam CT score was modeled with a three-knot restricted cubic spline. The corresponding complete-case estimate was 2.76 (95% CI, 2.24-3.40), and the estimate from the existing single-imputed working dataset was 2.86 (95% CI, 2.33-3.50). The direction and magnitude of association were consistent across missing-data approaches.
