# Supplementary material for: Admission TRIAGES score for unfavorable functional outcome after traumatic brain injury: association, prognostic performance, and comparison with the Glasgow coma scale
Source: Front Neurol. 2026 Oct 2;17:1963243. doi: 10.3389/fneur.2026.1963243 (PMC13671951; doi:10.3389/fneur.2026.1963243)
Supplement: Supplementary file 6 [file Supplementary_file_6.DOCX]

**Multivariable Association Models and Diagnostic Assessment**

**S3.1 Model specification and estimation**

The TRIAGES score was prespecified as the primary predictor and was not selected using univariable screening or penalized regression. Unfavorable functional status at discharge (GOS 1–3) was modeled using logistic regression. Model 1 was unadjusted. Model 2, the primary adjusted model, included sex, injury mechanism, the number of nonreactive pupils, and Rotterdam CT score. Model 3 additionally included admission blood glucose, white blood cell count, platelet count, and fibrinogen. Each model was fitted in 20 multiply imputed datasets, and coefficients and standard errors were combined using Rubin’s rules. The TRIAGES score was reported per 1-point and per 1-standard-deviation increase.

Continuous-variable functional forms were assessed using three-knot restricted cubic splines. TRIAGES and the four laboratory covariates showed no evidence of nonlinearity and were retained as linear terms. Rotterdam CT score showed evidence of nonlinearity; consequently, Models 2 and 3 used a restricted cubic spline with knots at scores 1, 2, and 5. The spline term is summarized using an overall pooled Wald test because its individual basis coefficients do not have a clinically meaningful odds-ratio interpretation.

**Supplementary Table S3A. Pooled logistic regression results for Models 1–3**

| **Predictor** | **Model 1, OR (95% CI)** | **P value** | **Model 2, OR (95% CI)** | **P value** | **Model 3, OR (95% CI)** | **P value** |
| --- | --- | --- | --- | --- | --- | --- |
| TRIAGES score, per 1-point increase | 3.13 (2.63–3.72) | <0.001 | 2.81 (2.29–3.45) | <0.001 | 2.85 (2.31–3.52) | <0.001 |
| Male sex (reference: female) | — | — | 1.64 (0.95–2.82) | 0.077 | 1.74 (0.99–3.04) | 0.053 |
| **Injury mechanism, overall** | — | — | — | 0.306 | — | 0.304 |
| Fall from height vs traffic accident | — | — | 1.91 (0.64–5.68) | 0.247 | 1.94 (0.63–5.99) | 0.252 |
| Ground-level fall vs traffic accident | — | — | 0.64 (0.24–1.71) | 0.374 | 0.65 (0.23–1.82) | 0.413 |
| Other vs traffic accident | — | — | 1.11 (0.53–2.34) | 0.779 | 1.24 (0.57–2.67) | 0.590 |
| Number of nonreactive pupils, per additional pupil | — | — | 1.69 (1.12–2.56) | 0.013 | 1.69 (1.11–2.57) | 0.015 |
| **Rotterdam CT score, overall (RCS; 2 df)** | — | — | RCS | 0.006 | RCS | 0.010 |
| Blood glucose, per 1 mmol/L | — | — | — | — | 1.11 (1.03–1.20) | 0.008 |
| White blood cell count, per 1 ×10⁹/L | — | — | — | — | 1.00 (0.96–1.05) | 0.916 |
| Platelet count, per 1 ×10⁹/L | — | — | — | — | 1.00 (0.99–1.00) | 0.139 |
| Fibrinogen, per 1 g/L | — | — | — | — | 0.80 (0.60–1.07) | 0.128 |

**Note: Model 1 was unadjusted. Model 2 was adjusted for sex, injury mechanism, number of nonreactive pupils, and Rotterdam CT score and was designated as the primary adjusted model. Model 3 additionally included blood glucose, white blood cell count, platelet count, and fibrinogen. Rotterdam CT score was modeled with an RCS; therefore, no single OR is reported for that term. Overall P values for multicategory and spline terms were obtained using pooled Wald tests. CI, confidence interval; OR, odds ratio; RCS, restricted cubic spline.**

**S3.2 Model diagnostic assessment**

Multicollinearity was assessed using term-level generalized variance inflation factors (GVIFs) in each imputed dataset. For multi-degree-of-freedom terms, GVIFs were transformed as GVIF^(1/[2 × df]). Functional-form diagnostics used pooled tests of the nonlinear spline component. Event sufficiency was summarized descriptively using the number of unfavorable outcomes per predictor parameter. Influence diagnostics were evaluated separately in each imputed dataset; screening thresholds were not used as automatic exclusion rules.

**Supplementary Table S3B. Multicollinearity diagnostics across the 20 imputed datasets**

| **Model** | **Term** | **df** | **Median GVIF (range)** | **Median adjusted GVIF (range)** | **Interpretation** |
| --- | --- | --- | --- | --- | --- |
| Model 2 | TRIAGES score | 1 | 1.74 (1.72–1.76) | 1.32 (1.31–1.33) | No problematic collinearity |
| Model 2 | Sex | 1 | 1.03 (1.03–1.04) | 1.02 (1.01–1.02) | No problematic collinearity |
| Model 2 | Injury mechanism | 3 | 1.06 (1.05–1.07) | 1.01 (1.01–1.01) | No problematic collinearity |
| Model 2 | Number of nonreactive pupils | 1 | 1.52 (1.48–1.54) | 1.23 (1.22–1.24) | No problematic collinearity |
| Model 2 | Rotterdam CT score (RCS) | 2 | 1.80 (1.77–1.85) | 1.16 (1.15–1.17) | No problematic collinearity |
| Model 3 | TRIAGES score | 1 | 1.79 (1.76–1.81) | 1.34 (1.33–1.35) | No problematic collinearity |
| Model 3 | Sex | 1 | 1.06 (1.05–1.06) | 1.03 (1.03–1.03) | No problematic collinearity |
| Model 3 | Injury mechanism | 3 | 1.09 (1.08–1.10) | 1.02 (1.01–1.02) | No problematic collinearity |
| Model 3 | Number of nonreactive pupils | 1 | 1.53 (1.49–1.55) | 1.24 (1.22–1.24) | No problematic collinearity |
| Model 3 | Rotterdam CT score (RCS) | 2 | 1.95 (1.92–2.00) | 1.18 (1.18–1.19) | No problematic collinearity |
| Model 3 | Blood glucose | 1 | 1.16 (1.14–1.17) | 1.08 (1.07–1.08) | No problematic collinearity |
| Model 3 | White blood cell count | 1 | 1.31 (1.30–1.33) | 1.14 (1.14–1.15) | No problematic collinearity |
| Model 3 | Platelet count | 1 | 1.15 (1.13–1.17) | 1.07 (1.06–1.08) | No problematic collinearity |
| Model 3 | Fibrinogen | 1 | 1.12 (1.11–1.13) | 1.06 (1.05–1.06) | No problematic collinearity |

**Note: For one-degree-of-freedom terms, the unadjusted GVIF is equivalent to the conventional VIF. The adjusted GVIF facilitates comparison across terms with differing degrees of freedom. All adjusted GVIF values were well below 2.24, the value corresponding to a VIF of 5 for a one-degree-of-freedom term.**

**Supplementary Table S3C. Assessment of linearity in the logit for continuous predictors**

| **Continuous predictor** | **Model assessed** | **RCS knots** | **P for nonlinearity** | **Median ΔAIC (range)** | **Final functional form** |
| --- | --- | --- | --- | --- | --- |
| TRIAGES score | Model 2 | 0, 2, 5 | 0.366 | 1.10 (1.02–1.31) | Linear |
| Rotterdam CT score | Model 2 | 1, 2, 5 | 0.006 | -6.21 (-7.47–-5.82) | Restricted cubic spline |
| Blood glucose | Model 3 | 5.74, 7.58, 12.1 | 0.316 | 0.93 (-0.01–1.53) | Linear |
| White blood cell count | Model 3 | 7.81, 13.465, 22.3 | 0.671 | 1.82 (1.70–1.89) | Linear |
| Platelet count | Model 3 | 149, 223, 305 | 0.605 | 1.72 (1.20–1.97) | Linear |
| Fibrinogen | Model 3 | 1.51, 2.32, 3.62 | 0.440 | 1.42 (0.35–1.91) | Linear |

**Note: Three-knot RCS functions used the 10th, 50th, and 90th percentiles. ΔAIC was calculated as AIC for the spline model minus AIC for the corresponding linear model within each imputed dataset; negative values favor the spline model. Rotterdam CT score showed evidence of nonlinearity and was modeled using an RCS in the final Models 2 and 3. AIC, Akaike information criterion; RCS, restricted cubic spline.**

**Supplementary Table S3D. Outcome events relative to model parameters**

| **Model** | **Patients, n** | **Unfavorable outcomes, n** | **Predictor parameters, n** | **Events per parameter** | **Assessment** |
| --- | --- | --- | --- | --- | --- |
| Model 1 | 753 | 181 | 1 | 181.0 | Adequate relative to prespecified model complexity |
| Model 2 | 753 | 181 | 8 | 22.6 | Adequate relative to prespecified model complexity |
| Model 3 | 753 | 181 | 12 | 15.1 | Adequate relative to prespecified model complexity |

**Note: Parameter counts exclude the intercept and count the three injury-mechanism contrasts separately. The Rotterdam CT spline contributed two parameters in Models 2 and 3. Events per parameter are provided as a descriptive measure and do not replace internal validation, which is planned using bootstrap resampling.**

**Supplementary Table S3E. Model convergence and influence diagnostics across imputed datasets**

| **Model** | **Converged datasets** | **Separation detected** | **Median maximum Cook’s distance (range)** | **Median maximum leverage (range)** | **Median n with Cook’s D > 4/n (range)** | **Database decision** |
| --- | --- | --- | --- | --- | --- | --- |
| Model 1 | 20/20 | 0/20 | 0.034 (0.034–0.034) | 0.006 (0.006–0.006) | 65 (65–65) | No observation removed solely on influence diagnostics |
| Model 2 | 20/20 | 0/20 | 0.028 (0.025–0.030) | 0.073 (0.070–0.088) | 57 (54–59) | No observation removed solely on influence diagnostics |
| Model 3 | 20/20 | 0/20 | 0.023 (0.021–0.066) | 0.199 (0.171–0.216) | 63 (58–67) | No observation removed solely on influence diagnostics |

**Note: All models converged in all imputed datasets, and no complete or quasi-complete separation was detected. Cook’s distance >4/n was used as a screening flag rather than an exclusion criterion. Maximum Cook’s distances remained below 0.07 across all models and imputations. No patient record was deleted solely because of residual, leverage, or influence diagnostics.**

**S3.3 Diagnostic summary**

The prespecified association between TRIAGES and unfavorable discharge GOS was robust across the crude, primary adjusted, and extended models. No problematic multicollinearity was identified. The linearity assumption was supported for TRIAGES and the laboratory covariates, whereas Rotterdam CT score required nonlinear modeling. The number of unfavorable outcomes was adequate relative to the number of prespecified predictor parameters. All models converged across the 20 imputed datasets, and influence diagnostics did not support excluding any observation from the primary analyses.

**S3.4 Abbreviations**

AIC, Akaike information criterion; CI, confidence interval; CT, computed tomography; df, degrees of freedom; GOS, Glasgow Outcome Scale; GVIF, generalized variance inflation factor; OR, odds ratio; RCS, restricted cubic spline; SD, standard deviation; TRIAGES, Trauma Rating Index in Age, Glasgow Coma Scale, Respiratory rate and Systolic blood pressure; VIF, variance inflation factor.
