# Supplementary material for: Admission TRIAGES score for unfavorable functional outcome after traumatic brain injury: association, prognostic performance, and comparison with the Glasgow coma scale
Source: Front Neurol. 2026 Oct 2;17:1963243. doi: 10.3389/fneur.2026.1963243 (PMC13671951; doi:10.3389/fneur.2026.1963243)
Supplement: Supplementary file 7 [file Supplementary_file_7.DOCX]

**Supplementary Table S5 Bootstrap internal validation of the TRIAGES score model in the single-imputation analytic dataset**

| **Performance measure** | **Apparent estimate** | **Mean signed optimism** | **Optimism-corrected estimate** |
| --- | --- | --- | --- |
| Area under the ROC curve | 0.905 | 0.000 | 0.905 |
| Brier score | 0.101 | -0.001 | 0.102 |
| Calibration intercept | 0.000 | -0.008 | 0.008 |
| Calibration slope | 1.000 | 0.002 | 0.998 |

Internal validation was performed using 1,000 nonparametric bootstrap resamples of the single-imputation analytic cohort (n = 753; unfavorable discharge GOS events, n = 181). The model contained the TRIAGES score as the sole predictor and corresponded to the TRIAGES-alone ROC, calibration, and decision-curve analyses (apparent AUC, 0.905). In each bootstrap resample, the intercept and TRIAGES coefficient were re-estimated, and model performance was evaluated in both the bootstrap sample and the original cohort. Mean signed optimism was calculated as bootstrap-sample performance minus performance when the bootstrap-fitted model was evaluated in the original cohort. Optimism-corrected estimates were calculated as the apparent estimate minus the mean signed optimism. Small negative signed optimism values for AUC or Brier score reflect Monte Carlo variation and indicate essentially negligible overfitting. Missing-data imputation was completed once before bootstrapping and was not repeated within each bootstrap resample; therefore, these results represent internal validation of the single-imputation TRIAGES score model and not bootstrap validation of the pooled MICE analysis.

Abbreviations: AUC, area under the receiver operating characteristic curve; GCS, Glasgow Coma Scale; GOS, Glasgow Outcome Scale; MICE, multiple imputation by chained equations; ROC, receiver operating characteristic; TRIAGES, Trauma Rating Index in Age, Glasgow Coma Scale, Respiratory rate and Systolic blood pressure.
